# Lfng-expressing centroacinar cell is a unique cell-of-origin for p53 deficient pancreatic cancer

**DOI:** 10.1038/s41388-024-03226-7

**Published:** 2024-11-15

**Authors:** Wen-Cheng Chung, Shubing Zhang, Azeddine Atfi, Keli Xu

**Affiliations:** 1 Department of Cell and Molecular Biology, University of Mississippi Medical Center, Jackson, 39216 USA; 2 Cancer Center and Research Institute, University of Mississippi Medical Center, Jackson, 39216 USA; 3 Department of Cell Biology, School of Life Sciences, Central South University, Changsha, 410013 China; 4 Department of Biochemistry and Molecular Biology, Massey Cancer Center, Virginia Commonwealth University, Richmond, VA 23298 USA

**Keywords:** Oncogenes, Cell signalling, Cancer stem cells

## Abstract

Pancreatic ductal adenocarcinoma (PDAC) is one of the most lethal malignancies with limited understanding of etiology. Studies in mice showed that both acinar and ductal cells of the pancreas can be targeted by combination of oncogenic Kras and p53 mutations to form PDAC. How the transforming capacities of pancreatic cells are constrained, and whether a subset of cells could serve as a prime target for oncogenic transformation, remain obscure. Here we report that expression of a Notch modulator, Lunatic Fringe (Lfng), is restricted to a limited number of cells with centroacinar location and morphology in the adult pancreas. Lfng-expressing cells are preferentially targeted by oncogenic Kras along with p53 deletion to form PDAC, and deletion of Lfng blocks tumor initiation from these cells. Notch3 is a functional Notch receptor for PDAC initiation and progression in this context. Lfng is upregulated in acinar- and ductal-derived PDAC and its deletion suppresses these tumors. Finally, high LFNG expression is associated with high grade and poor survival in human patients. Taken together, Lfng marks a centroacinar subpopulation that is uniquely susceptible to oncogenic transformation when p53 is lost, and Lfng functions as an oncogene in all three lineages of the exocrine pancreas.

## Introduction

Pancreatic ductal adenocarcinoma (PDAC) accounts for more than 90% of pancreatic cancer cases. It is developed in the exocrine pancreas and is one of the most lethal malignancies due to the lack of early diagnosis and poor response to most therapies. Genomic analyses have identified major genetic alterations associated with the initiation and progression of PDAC, including activating mutations in *KRAS* and mutations in the *TP53* tumor suppressor gene, which are detected in 94 and 64% of PDAC patients, respectively [1, 2]. Mouse models with genetic alterations similar to those found in PDAC patients have been generated to recapitulate pathologic changes, and to identify additional genes critically involved in PDAC pathogenesis. Among those, the Notch signaling pathway was found to be upregulated in preneoplastic lesions as well as in invasive tumors of the pancreas in both mice and humans [3–10], and targeting Notch2/Notch3 inhibited patient-derived xenograft tumor growth and decreased frequency of tumor-initiating cells [11]. However, the role of Notch signaling in PDAC appears contentious. Notch2 was required for pancreatic intraepithelial neoplasia (PanIN) progression and development of PDAC [8], whereas both loss- and gain-of-function of Notch1 rendered acinar cells more susceptible to Kras-induced transformation [3, 12, 13]. The Notch downstream target Hes1 was shown to restrict tumor development in a PDAC model with oncogenic Kras activated in the pancreas starting from embryonic stage [14]. Paradoxically, ablation of Hes1 completely blocked PDAC formation in mice where expression of Kras^G12D^ and Trp53^R172H^ was induced in acinar cells of the adult pancreas [15]. A comprehensive understanding of the complex roles of Notch in PDAC pathogenesis is therefore a critical gap in the development of potential Notch-targeted therapy for this disease.

The Fringe family of β3N-acteylglucosaminyl-transferases is known to modify EGF repeats in the extracellular domains of Notch, thereby modulating the ligand binding and activation of the Notch receptors [16–20]. Interestingly, Lunatic Fringe (Lfng) is selectively expressed in stem/progenitor-like cells in adult tissues including the mammary gland, hippocampus, and small intestine [21–23]. In the pancreas, Lfng expression is co-localized with the acinar differentiation determinant Ptf1a during embryonic development [24], and is restricted to a small number of cells in the exocrine compartment of adult pancreas [10]. Crossing conditional knockout of *Lfng* into *Kras*^*G12D*^*;Pdx1-Cre* mice resulted in accelerated PDAC development, suggesting a tumor-suppressive role for Lfng in this context [10]. However, Kras^G12D^ is activated in all pancreatic lineages starting from embryonic stage in these mice, and deletion of Lfng during embryonic development may cause defective acinar differentiation and thereby favor Kras-induced PanIN initiation and progression. Intriguingly, LFNG expression in PDAC cell lines as a group is the second highest among all cancer cell lines representing 29 tissues (Cancer Cell Line Encyclopedia). A recent study found that LFNG amplification was significantly increased in human PDAC, and high expression of LFNG was associated with poor overall survival [25]. LFNG overexpression promoted proliferation and invasion of a PDAC cell line Panc-1 [25]. Conversely, inactivating LFNG in Panc-1 impaired proliferation and migration [10, 26], and low LFNG expression predicted better overall survival in PDAC patients [26]. These findings suggest an oncogenic role for LFNG in human PDAC, contradictory to our previous study using the *Kras*^*G12D*^*;Pdx1-Cre* mouse model.

In this study, we characterized Lfng expression and function in the adult mouse pancreas by lineage tracing of *Lfng*-expressing cells under normal physiological condition, following exocrine injury, and during oncogenic transformation induced by Kras and p53 mutations, with or without a functional Lfng. We identified a centroacinar subpopulation as a unique cell-of-origin for PDAC with p53 loss. We also revealed an oncogenic role of Lfng in both initiation and progression of PDAC from three distinct cells-of-origin in mice, corroborating the genomic data and studies in human PDAC.

## Results

### Lfng expression in the adult pancreas is restricted to the centroacinar compartment

**Fig. 1:**
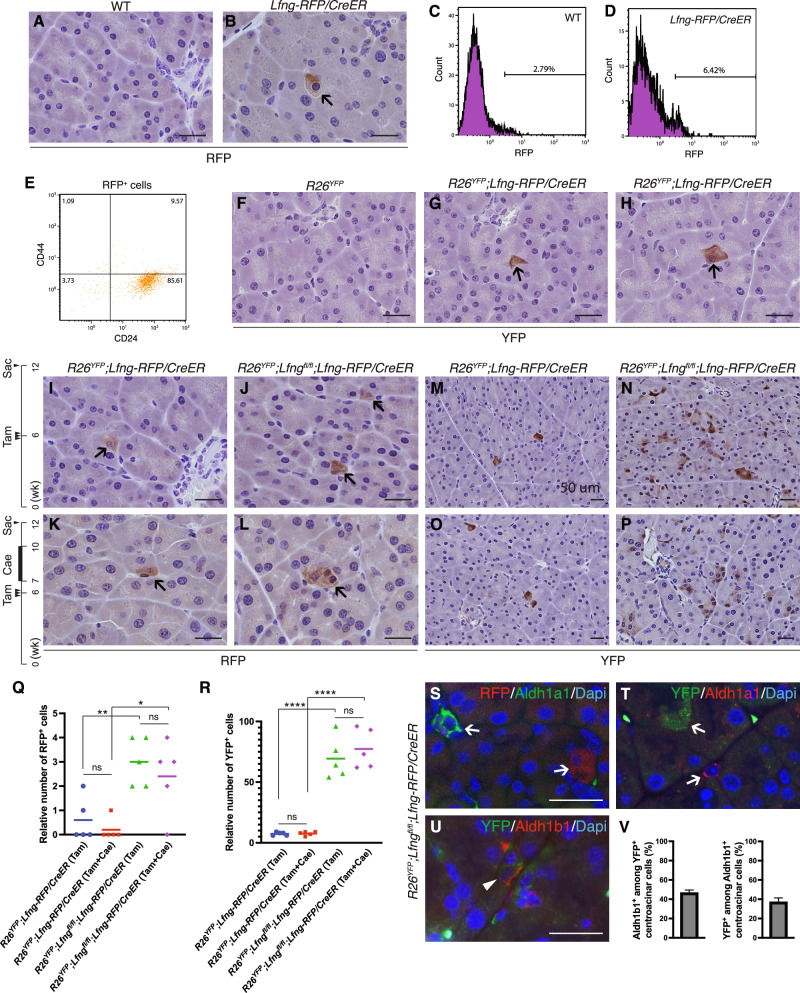
Lfng-expressing cells and their descendants in adult mouse pancreas. **A**, **B** Anti-RFP immunostaining in the pancreas from wild type and *Lfng-RFP/CreER* mice at 3 months of age. Arrow: positive staining in a centroacinar cell. Representative flow cytometry analysis for RFP^+^ cells in lineage-depleted pancreatic cells from adult wild type (**C**) and *Lfng-RFP/CreER* (**D**, **E**) mice. **F–H** Anti-YFP immunostaining in the pancreas from *R26*^*YFP*^ and *R26*^*YFP*^*;Lfng-RFP/CreER* mice 6 weeks after tamoxifen induction at 6 weeks of age. Arrows: positive staining in a centroacinar cell (**G**) and acinar cell (**H**). **I–L** Anti-RFP immunostaining in the pancreas from *R26*^*YFP*^*;Lfng-RFP/CreER* and *R26*^*YFP*^*;Lfng*^*fl/fl*^*;Lfng-RFP/CreER* mice 6 weeks after tamoxifen induction at 6 weeks of age, with or without caerulein treatment, which started at one week post-tamoxifen and lasted for 3 weeks. Arrows: positive staining in centroacinar cells. **M–P** Anti-YFP immunostaining in the pancreas from *R26*^*YFP*^*;Lfng-RFP/CreER* and *R26*^*YFP*^*;Lfng*^*fl/fl*^*;Lfng-RFP/CreER* mice 6 weeks after tamoxifen induction, with or without caerulein treatment. **Q** Quantitation of RFP^+^ cells depicted in I-L. *p < 0.05; **p < 0.01; ns: non-significance. **R** Quantitation of YFP^+^ cells depicted in M-P. ****p < 0.0001; ns: non**-**significance. **S** Double immunofluorescent staining for RFP (red) and Aldh1a1 (green) in *R26*^*YFP*^*;Lfng*^*fl/fl*^*;Lfng-RFP/CreER* pancreas. Arrows: an Aldh1a1^+^ cell (left) and an RFP^+^ cell (right). **T** Double immunofluorescent staining for YFP (green) and Aldh1a1 (red) in *R26*^*YFP*^*;Lfng*^*fl/fl*^*;Lfng-RFP/CreER* pancreas. Arrows: an YFP^+^ cell (top) and an Aldh1a1^+^ cell (bottom). **U** Double immunofluorescent staining for YFP (green) and Aldh1b1 (red) in *R26*^*YFP*^*;Lfng*^*fl/fl*^*;Lfng-RFP/CreER* pancreas. Arrowhead: co-staining of Aldh1b1 and YFP in a centroacinar cell. **V** Quantitation of cells with co-staining of YFP and Aldh1b1. Scale bars: 25 μm.

To characterize Lfng-expressing cells in the adult pancreas, we made use of a *Lfng-RFP/CreERT2* mouse strain expressing RFP and tamoxifen-inducible Cre under the *Lfng* promoter. Anti-RFP immunostaining in *Lfng-RFP/CreERT2* mice showed similar but more restricted *Lfng* expression in the exocrine pancreas compared to X-gal staining in *Lfng*^*lacZ*^ knock-in mice [10]. The vast majority of RFP^+^ cells are located near the centroacinar compartment and display centroacinar rather than acinar cell morphology (Fig. 1A, B). Bearing CD24^+^CD44^-^ surface marker, RFP^+^ cells account for about 3% of lineage depleted pancreatic cells (Fig. 1C–E). We performed lineage tracing for *Lfng*-expressing cells. While anti-YFP staining was completely negative in *R26*^*YFP*^ pancreas (Fig. 1F), *R26*^*YFP*^*;Lfng-RFP/CreERT2* pancreas showed YFP^+^ cells (descendants of *Lfng*-expressing cell) either remaining centroacinar (Fig. 1G) or becoming acinar (Fig. 1H) under homeostatic conditions. Quantitative lineage tracing with or without caerulein treatment showed comparable number of RFP^+^ cells as well as YFP^+^ cells, suggesting that caerulein-induced exocrine injury did not alter self-renewal or differentiation of Lfng-expressing cells. Next, we tested the effect of Lfng deletion on the fate of *Lfng*-expressing cells. *R26*^*YFP*^*;Lfng*^*fl/fl*^*;Lfng-RFP/CreERT2* mice exhibited significantly increased number of RFP^+^ cells and almost a 10-fold increase of YFP^+^ cells compared to the *R26*^*YFP*^*;Lfng-RFP/CreERT2* mice, irrespective of caerulein treatment (Fig. 1I–R). Many of the YFP^+^ cells in *R26*^*YFP*^*;Lfng*^*fl/fl*^*;Lfng-RFP/CreERT2* mice exhibited acinar location and morphology (Fig. 1N, P). Thus, deletion of Lfng appeared to enhance proliferation and differentiation of *Lfng*-expressing centroacinar cells to acinar cells. Progenitor subpopulations in the centroacinar and terminal duct cells express high level of ALDH1 enzymatic activity [27, 28]. We found no overlapping of either RFP or YFP with Aldh1a1 (Fig. 1S, T), though co-staining of YFP and Aldh1b1 was observed in a subset of centroacinar cells in *R26*^*YFP*^*;Lfng*^*fl/fl*^*;Lfng-RFP/CreERT2* mice (Fig. 1U, V). Interestingly, Lfng-expressing cells express high level of CD24 (Fig. 1E), a surface marker for pancreatic progenitors identified using human embryonic stem cells [29]. Additionally, CD24^high^ but not CD24^low^ subpopulation in the mouse pancreatic ducts was able to form organoids [30]. These results suggest that *Lfng*-expressing cells may represent a progenitor subpopulation in the centroacinar compartment.

### Lfng-expressing centroacinar cell can be targeted to form PanIN and progress to PDAC

**Fig. 2:**
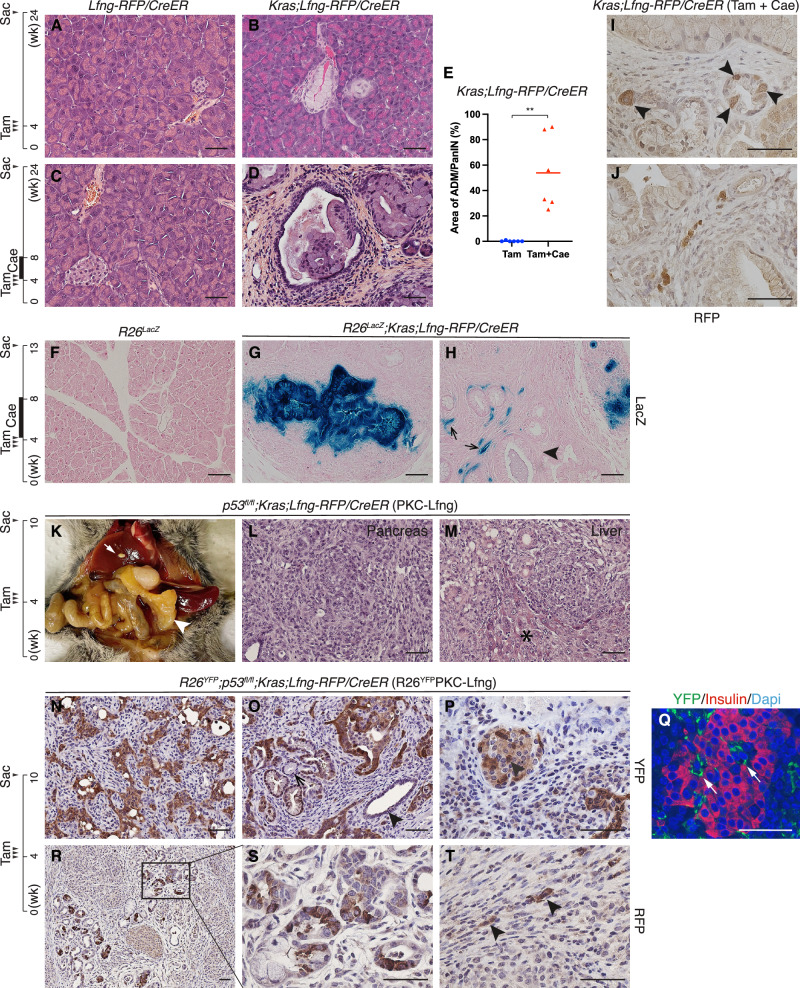
Lfng-expressing cells in the adult pancreas can be targeted to form PanIN and PDAC. **A–D** Hematoxylin and eosin staining of pancreas from *Lfng-RFP/CreER* and *Kras;Lfng-RFP/CreER* mice 20 weeks after tamoxifen injection at 4 weeks of age, with or without 4 weeks of daily caerulein treatment immediately following tamoxifen administration. **E** Quantification of ADM/PanIN lesions in *Kras;Lfng-RFP/CreER* mice depicted in (**B**, **D**). **p < 0.01. **F–H** X-Gal staining of the pancreas from *R26*^*LacZ*^ and *R26*^*LacZ*^*;Kras;Lfng-RFP/CreER* mice at 9 weeks after tamoxifen injection at 4 weeks of age. These mice also received 4 weeks of daily caerulein treatment immediately following tamoxifen administration. Arrows: positive staining in the stroma. Arrowhead: pseudo-PanIN. **I**, **J** Anti-RFP immunostaining in the pancreas from *Kras;Lfng-RFP/CreER* mice 16 weeks after tamoxifen injection followed by 4 weeks of caerulein treatment. **K** Representative gross pathology of the pancreatic tumor in *p53*^*fl/fl*^*;Kras;Lfng-RFP/CreER* (PKC-Lfng) mice at 7 weeks post-tamoxifen. Arrowhead: tumor of the entire pancreas. Arrow: metastatic growth on the liver. **L**, **M** Histology of the pancreatic tumor and liver metastasis shown in (K). * area of hepatocytes. **N**–**P** Anti-YFP immunostaining in the pancreas of 10-week-old *R26*^*YFP*^*;p53*^*fl/fl*^*;Kras;Lfng-RFP/CreER* (R26^YFP^PKC-Lfng) mice treated with tamoxifen at 4 weeks old. Arrow: YFP-negative PanIN-like lesion. Arrowhead in O: YFP-negative native duct. Arrowhead in P: YFP-positive cell within an islet. **Q** Double immunofluorescent staining for insulin (red) and YFP (green) in the R26^YFP^PKC-Lfng pancreas. Arrows: YFP-positive cells. **R–T** Anti-RFP immunostaining in pancreatic tumors from R26^YFP^PKC-Lfng mice. S is high-magnification image of the square area in R. Arrows: RFP-positive tumor cells in poorly differentiated area. Scale bars: 50 μm.

Stem/progenitor-like cells are thought to be the cellular origin for pancreatic tumors. We wondered whether Lfng-expressing cells could serve as a cell-of-origin for PDAC. *Lfng-RFP/CreERT2*-directed Kras^G12D^ alone rarely caused PanIN, but in conjunction with caerulein treatment, resulted in extensive PanIN (Fig. 2A–E). PanIN lesions in *R26*^*lacZ*^*;Kras;Lfng-RFP/CreERT2* mice stained positive for LacZ, confirming they were derived from Lfng-expressing cells (Fig. 2G). LacZ^+^ stromal cells (arrows in Fig. 2H) may represent descendants from Lfng-expressing cells that have undergone epithelial-mesenchymal transition (EMT) or PanIN-associated fibroblasts that have turned on Lfng expression, whereas LacZ^−^ PanIN-like lesions (arrowhead in Fig. 2H) could be pseudo-PanIN lesions induced by caerulein. RFP as a surrogate for Lfng expression was detected in a subset of PanIN cells (Fig. 2I) and in limited number of stromal cells (Fig. 2J). Next, we generated *p53*^*fl/fl*^*;Kras;Lfng-RFP/CreER* (hereinafter referred to as PKC-Lfng) mice to drive Kras^G12D^ expression coupled with p53 deletion in Lfng-expressing cells. These mice developed pancreatic tumors with metastasis in less than 2 months following tamoxifen induction (Fig. 2K–M). Anti-YFP staining of the *R26*^*YFP*^*;p53*^*fl/fl*^*;Kras;Lfng-RFP/CreER* (R26^YFP^PKC-Lfng) pancreas was positive in all tumor cells (Fig. 2N) and negative in native duct (arrowhead in Fig. 2O), confirming that tumor cells had originated from Lfng-expressing cells. There were YFP^−^ PanIN-like lesions (arrow in Fig. 2O), likely representing pseudo-PanIN lesions induced by adjacent PDAC [31]. YFP staining was noted within the islet in R26^YFP^PKC-Lfng mice (Fig. 2P), but not in *R26*^*YFP*^*;Lfng-RFP/CreER* mice (data not shown). These cells were insulin-negative (Fig. 2Q), suggesting tumor cell invasion into the islet rather than conversion of targeted cell to endocrine cell. Anti-RFP staining revealed Lfng expression in PanIN and ductal tumor cells, and occasionally in mesenchymal-like tumor cells (Fig. 2R–T).

We treated PKC-Lfng mice with caerulein immediately following tamoxifen induction at one month of age. Almost two-thirds of PKC-Lfng mice died during one month of daily caerulein treatment. Surprisingly, PKC-Lfng mice that survived the caerulein treatment lived much longer compared to those without caerulein treatment (Supplemental Fig. 1A). Congruently, 3 out of 7 such mice showed very few PanINs (no PDAC) at 3 months post tamoxifen (Supplemental Fig. 1B). Of note, caerulein treatment started at 1 month post tamoxifen, a time point when PanIN had formed, had no effect on tumor onset (Supplemental Fig. 1C). Thus, while caerulein treatment accelerates Kras-induced PanIN from p53-intact Lfng-expressing cells, it may prevent PanIN formation from p53-deficient Lfng-expressing cells.

We also generated *p53*^*R172H*^*;Kras;Lfng-RFP/CreER* mice to drive Kras^G12D^ and p53^R172H^ expression in Lfng-expressing cells. These mice developed very few PanIN without caerulein treatment (Supplemental Fig. 2A–C) but succumbed to PDAC with caerulein treatment following tamoxifen induction (Supplemental Fig. 2D–H). Descendants of Lfng-expressing cells in these mice were seen in normal acini, PanIN, and invasive tumors (Supplemental Fig. 2J–K). Like PKC-Lfng mice, this model showed Lfng expression in a few PanINs and limited number of tumor cells (Supplemental Fig. 2L–N). Collectively, these results demonstrate that Lfng-expressing cells in adult pancreas are readily targeted by combinatory Kras and p53 mutations to form PDAC.

### Lfng-expressing centroacinar cell serves as a unique cell-of-origin for loss-of-p53 PDAC

**Fig. 3:**
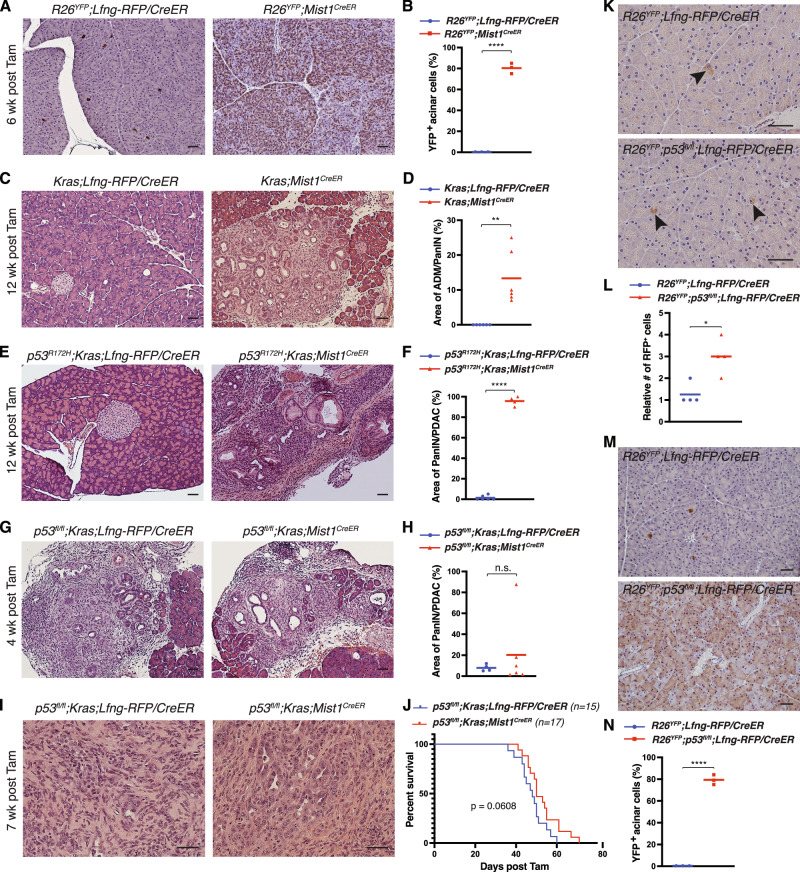
Lfng-expressing cells are preferentially targeted by combination of Kras^G12D^ and p53 loss-of-function mutations. **A** Anti-YFP immunostaining in the pancreas from *R26*^*YFP*^*;Lfng-RFP/CreER* and *R26*^*YFP*^*;Mist1*^*CreER/+*^ mice 6 weeks after tamoxifen injection. **B** Quantitation of YFP^+^ cells depicted in (**A**), presented as the percentage of YFP^+^ cells among all cells in the acinar compartment. ****p < 0.0001. **C** Representative photomicrographs of the pancreas from *Kras;Lfng-RFP/CreER* and *Kras;Mist1*^*CreER/+*^ mice at 12 weeks after tamoxifen injection. **D** Quantitation of ADM and PanIN lesions in *Kras;Lfng-RFP/CreER* and *Kras;Mist1*^*CreER/+*^ mice at 12 weeks post-tamoxifen, presented as the percentage of total area affected by ADM and PanIN lesions. **p < 0.01. **E** Representative photomicrographs of the pancreas from *p53*^*R172H*^*;Kras;Lfng-RFP/CreER* and *p53*^*R172H*^*;Kras;Mist1*^*CreER/+*^ mice at 12 weeks after tamoxifen injection. **F** Quantitation of PanIN and PDAC lesions in *p53*^*R172H*^*;Kras;Lfng-RFP/CreER* and *p53*^*R172H*^*;Kras;Mist1*^*CreER/+*^ mice at 12 weeks post-tamoxifen, presented as the percentage of total area affected by PanIN and PDAC lesions. ****p < 0.0001. **G** Representative photomicrographs of the pancreas from *p53*^*fl/fl*^*;Kras;Lfng-RFP/CreER* and *p53*^*fl/fl*^*;Kras;Mist1*^*CreER/+*^ mice at 4 weeks after tamoxifen injection. **H** Quantitation of PanIN and PDAC lesions in *p53*^*fl/fl*^*;Kras;Lfng-RFP/CreER* and *p53*^*fl/fl*^*;Kras;Mist1*^*CreER/+*^ mice at 4 weeks post-tamoxifen, presented as the percentage of total area affected by PanIN and PDAC lesions. ns: non-significance. **I** Representative histology of the pancreatic tumors from *p53*^*fl/fl*^*;Kras;Lfng-RFP/CreER* and *p53*^*fl/fl*^*;Kras;Mist1*^*CreER/+*^ mice at 7 weeks after tamoxifen injection. **J** Kaplan-Meier survival analysis for *p53*^*fl/fl*^*;Kras;Lfng-RFP/CreER* and *p53*^*fl/fl*^*;Kras;Mist1*^*CreER/+*^ mice following tamoxifen injection. Log-rank test *p* = 0.0608. **K**, **L** Anti-RFP immunostaining and quantitation of RFP^+^ cells in the pancreas from *R26*^*YFP*^*;Lfng-RFP/CreER* and *R26*^*YFP*^*;p53*^*fl/fl*^*;Lfng-RFP/CreER* mice 6 weeks after tamoxifen injection at 6 weeks of age. *p < 0.05. **M**, **N** Anti-YFP immunostaining and quantitation of YFP^+^ cells in the pancreas from *R26*^*YFP*^*;Lfng-RFP*^*/*^*CreER* and *R26*^*YFP*^*;p53*^*fl/fl*^*;Lfng-RFP/CreER* mice 6 weeks after tamoxifen injection at 6 weeks of age. **** *p* < 0.0001. Scale bars: 50 μm.

As noted above, Lfng-expressing cells account for only 3% of lineage depleted pancreatic cells. *Lfng-RFP/CreER*-mediated recombination occurs in less than 1% of pancreatic cells, compared to 80% recombination efficiency of *Mist1*^*CreER/+*^ in acinar cells (Fig. 3A, B). Despite the very low percentage of pancreatic cells being targeted, PKC-Lfng mice developed PDAC shortly after tamoxifen induction, suggesting that Lfng-expressing cells may serve as an important cell-of-origin for PDAC. We compared tumor development in mice targeted with the same set of oncogenic mutations directed by *Lfng-RFP/CreER* versus *Mist1*^*CreER/+*^. Kras^G12D^ alone readily induced PanIN under *Mist1*^*CreER/+*^, but almost none under *Lfng-RFP/CreER* (Fig. 3C, D). Combination of Kras^G12D^ and p53^R172H^ under *Mist1*^*CreER/+*^ caused formation of PanIN/PDAC affecting almost entire pancreas but resulted in very few precursor lesions when driven by *Lfng-RFP/CreER* (Fig. 3E, F). Strikingly, combination of Kras^G12D^ and p53 deletion through *Lfng-RFP/CreER* induced rapid PanIN development and progression to PDAC, comparable to those through *Mist1*^*CreER/+*^ (Fig. 3G–I), and even slightly shortened survival (Fig. 3J). *Mist1* is expressed in acinar but not centroacinar cells [32], whereas Lfng expression is mostly confined to the centroacinar compartment. These results indicate that Lfng marks a centroacinar subpopulation serving as a unique cell-of-origin for PDAC when targeted by oncogenic Kras coupled with p53 deletion. To understand why Lfng-expressing cells are uniquely susceptible to transformation when p53 is lost, we examined the effect of p53 deletion on the fate of Lfng-expressing cells by staining for RFP and YFP in *R26*^*YFP*^*;Lfng-RFP/CreERT2* versus *R26*^*YFP*^*;p53*^*fl/fl*^*;Lfng-RFP/CreERT2* following tamoxifen induction. Deleting p53 in *Lfng*-expressing cells caused a modest expansion of this population (Fig. 3K, L) and a drastic increase of their progenies (Fig. 3M, N), supporting that p53 constrains the propagation of *Lfng*-expressing cells in the adult pancreas.

### PDAC derived from Lfng-expressing cell is enriched in Aldh1^+^ progenitor-like cells and contains endocrine tumor cells

**Fig. 4:**
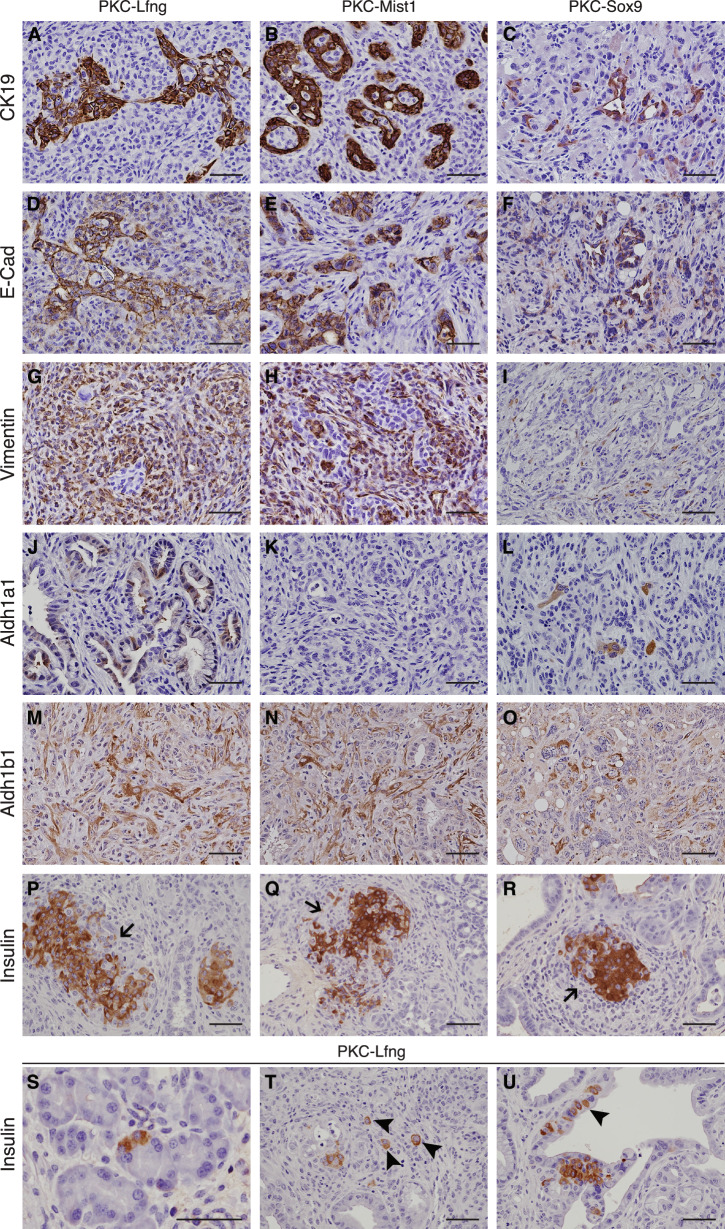
Immunohistochemical characterization of pancreatic tumors derived from centroacinar, acinar, and ductal cells. **A–C** Immunostaining for cytokeratin 19 (CK19) in pancreatic tumors from *p53*^*fl/fl*^*;Kras;Lfng-RFP/CreER* (PKC-Lfng), *p53*^*fl/fl*^*;Kras;Mist1*^*CreER/+*^ (PKC-Mist1), and *p53*^*fl/fl*^*;Kras;Sox9-CreER* (PKC-Sox9) mice. **D–F** Immunostaining for E-cadherin in pancreatic tumors from PKC-Lfng, PKC-Mist1, and PKC-Sox9 mice. **G–I** Immunostaining for vimentin in pancreatic tumors from PKC-Lfng, PKC-Mist1, and PKC-Sox9 mice. **J–L** Immunostaining for Aldh1a1 in pancreatic tumors from PKC-Lfng, PKC-Mist1, and PKC-Sox9 mice. **M–O** Immunostaining for Aldh1b1 in pancreatic tumors from PKC-Lfng, PKC-Mist1, and PKC-Sox9 mice. **P–R** Anti-insulin immunostaining in pancreatic tumors from PKC-Lfng, PKC-Mist1, and PKC-Sox9 mice. Arrows: insulin-negative area within the islet. **S–U** Anti-insulin immunostaining in the pancreas from PKC-Lfng mice. Arrows: insulin-positive solitary tumor cells (**T**) and ductal tumor cells (**U**). Scale bars: 50 μm.

We performed immunohistochemistry on PKC-Lfng tumors side-by-side with acinar-derived *p53*^*fl/fl*^*;Kras;Mist1*^*CreER*^ (hereinafter referred to as PKC-Mist1) and ductal-derived *p53*^*fl/fl*^*;Kras;Sox9-CreER* (hereinafter referred to as PKC-Sox9) tumors. Both PKC-Lfng and PKC-Mist1 tumors are positive for ductal marker CK19 and epithelial marker E-Cadherin along with high expression of vimentin, whereas PKC-Sox9 tumors exhibit lower expression of these markers (Fig. 4A–I). Tumors derived from Lfng-expressing centroacinar cells may be enriched in progenitor-like cells. Indeed, PKC-Lfng tumors showed more Aldh1a1^+^ cells compared to PKC-Sox9 tumors, whereas PKC-Mist1 tumors were completely negative for Aldh1a1 (Fig. 4J–L). For comparison, all three tumor types showed robust Aldh1b1 expression (Fig. 4M–O). Consistent with our previous finding that pancreatic cancer triggers selective depletion of β-cells [33], immunostaining revealed various insulin-negative spaces within the islets in three PDAC models (Fig. 4P–R). Interestingly, pancreas of PKC-Lfng mice contain morphologically normal acinar cells as well as tumor cells that express insulin (Fig. 4S–U), which are rarely seen in PKC-Mist1 and PKC-Sox9 mice. Lfng-expressing cells are CD24^+^ (Fig. 1E). CD24^+^, but not CD24^−^, human pancreatic progenitor cells can differentiate into insulin-producing cells [29]. These results suggest that Lfng-expressing centroacinar cells are multipotent and may adopt an endocrine fate under oncogenic transformation.

### Lfng is required for the initiation of PDAC from Lfng-expressing centroacinar cell

**Fig. 5:**
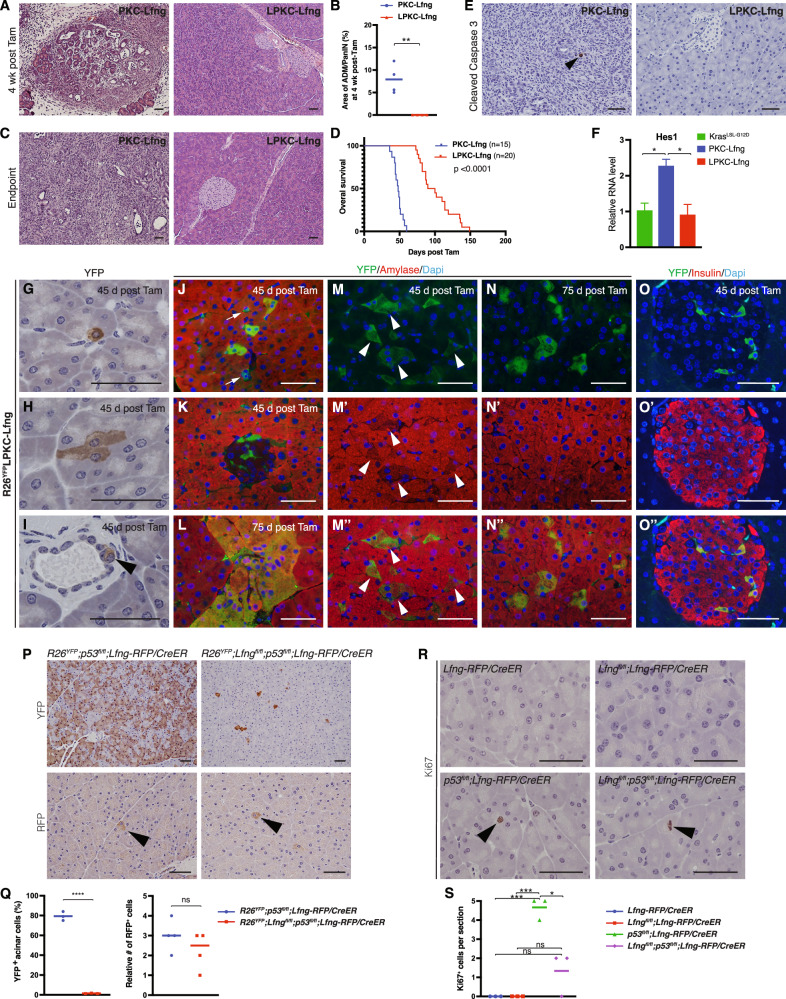
Deletion of Lfng blocked tumor initiation from Lfng-expressing pancreatic cells. **A**, **B** Representative photomicrographs of the pancreas from *p53*^*fl/fl*^*;Kras;Lfng-RFP/CreER* (PKC-Lfng) and *Lfng*^*fl/fl*^*;p53*^*fl/fl*^*;Kras;Lfng-RFP/CreER* (LPKC-Lfng) mice at 4 weeks post tamoxifen induction (**A**) and quantitation of ADM/PanIN lesions in these mice presented as the percentage of total area affected by precancerous lesions (**B**). **p < 0.01. **C** Representative photomicrographs of the pancreas from PKC-Lfng and LPKC-Lfng mice at the pathological endpoint. **D** Kaplan-Meier survival analysis for PKC-Lfng and LPKC-Lfng mice following tamoxifen injection. Log-rank test *p* < 0.0001. **E** Immunostaining for cleaved caspase 3 in the PKC-Lfng and LPKC-Lfng pancreas at 6 weeks post tamoxifen induction. Arrowhead: an apoptotic cell positive for cleaved caspase 3. **F** Relative mRNA level of *Hes1* in the *Kras*^*LSL-G12D*^, PKC-Lfng, and LPKC-Lfng pancreas, determined by quantitative RT-PCR. *p < 0.05. **G–I** Anti-YFP immunostaining in the pancreas of *R26*^*YFP*^*;Lfng*^*fl/fl*^*;p53*^*fl/fl*^*;Kras;Lfng-RFP/CreER* (R26^YFP^LPKC-Lfng) mice 45 days after tamoxifen injection. Arrowhead: YFP-positive cell in a normal duct. **J–L** Double immunofluorescence staining for amylase (red) and YFP (green) in the pancreas from R26^YFP^LPKC-Lfng mice at 45 and 75 days post-tamoxifen. Arrows in J: YFP^+^ centroacinar cells. **M-M”** Immunofluorescence staining for YFP (M), amylase (M’), and merged (M”) in the R26^YFP^LPKC-Lfng pancreas at 45 days post-tamoxifen. Arrowheads: YFP^+^ acinar cells showing lower amylase expression. **N-N”** Immunofluorescence staining for YFP (**N**), amylase (**N**’) and merged (**N**”) in the R26^YFP^LPKC-Lfng pancreas at 75 days post-tamoxifen. **O-O”** Immunofluorescence staining for YFP (**O**), insulin (**O**’), and merged (**O**”) in the R26^YFP^LPKC-Lfng pancreas at 45 days post-tamoxifen. **P** Anti-YFP and anti-RFP immunostaining in the pancreas of *R26*^*YFP*^*;p53*^*fl/fl*^*;Lfng-RFP/CreER* and *R26*^*YFP*^*;Lfng*^*fl/fl*^*;p53*^*fl/fl*^*;Lfng-RFP/CreER* mice 45 days after tamoxifen injection. Arrowheads: positive RFP staining in centroacinar cells. **Q** Quantitation of YFP^+^ cells and RFP^+^ cells depicted in (**P**). ****p < 0.0001. ns: non-significance. **R** Anti-Ki67 immunostaining in the pancreas of indicated genotypes at 45 days after tamoxifen injection. **S** Quantitation of Ki67^+^ cells depicted in (**R**). *p < 0.05, ***p < 0.001^,^ ns: non-significance. Scale bars: 50 μm.

To determine whether Lfng plays a role in PDAC arising from Lfng-expressing cells, we examined tumor development in *Lfng*^*fl/fl*^*;p53*^*fl/fl*^*;Kras;Lfng-RFP/CreER* (LPKC-Lfng) in comparison with PKC-Lfng mice. At 4 weeks post-tamoxifen, PKC-Lfng mice had developed PanIN lesions covering about 8% of the pancreatic tissue, whereas the pancreas from LPKC-Lfng mice was lesion-free (Fig. 5A, B). Consistent with this observation, LPKC-Lfng mice had significantly prolonged survival as compared to PKC-Lfng mice (Fig. 5D). At pathological endpoint, all PKC-Lfng mice had advanced PDAC, whereas the vast majority of LPKC-Lfng remained pancreatic lesion-free (Fig. 5C). Hemangioma-like lesions were found at various sites in LPKC-Lfng mice (data not shown), which may be the cause of morbidity. Since *Lfng* is expressed in venous endothelial cells [34] (Supplemental Fig. 4D), vascular lesions found in LPKC-Lfng mice were more likely primary tumors than metastasis from pancreas. Thus, deletion of Lfng blocks PDAC arising from Lfng-expressing centroacinar cells in adult mice. LPKC-Lfng pancreas stained negative for cleaved caspase 3, indicating that apoptosis was not the underlying mechanism of tumor suppression (Fig. 5E). Like *Kras;Mist1*^*CreER*^ [32], PKC-Lfng tumors showed upregulation of Hes1 compared to the pancreas from *Kras*^*LSL-G12D*^ control mice. Interestingly, LPKC-Lfng pancreas showed Hes1 expression comparable to that of *Kras*^*LSL-G12D*^ mice, suggesting that deletion of Lfng might revert Notch activation in PKC-Lfng pancreas (Fig. 5F). Next, we traced Lfng-expressing cells by anti-YFP staining in *R26*^*YFP*^*;Lfng*^*fl/fl*^*;p53*^*fl/fl*^*;Kras;Lfng-RFP/CreER* (R26^YFP^LPKC-Lfng) mice. The vast majority of YFP^+^ cells were found in the acinar compartment, resembling centroacinar and acinar cells (Fig. 5G, H), while rare YFP^+^ cells were detected in morphologically normal duct (Fig. 5I). Immunofluorescence staining showed many YFP^+^ cells co-expressing the acinar marker amylase (Fig. 5J, L), whereas those remained in centroacinar location were amylase^−^ (arrows in Fig. 5J). Interestingly, a few YFP^+^ cells residing in the islet expressed insulin (Fig. 5K, O-O”), suggesting conversion of Lfng-expressing cells to β-cells. We also noted YFP^+^ acinar-like cells with lower amylase expression compared to genuine acinar cells at early (Fig. 5M-M”), but not late (Fig. 5N-N”), time points, suggesting progressive transition to acinar cells. Taken together, inactivation of Lfng drives Kras^G12D^,p53^Deletion^-targeted centroacinar cells to adopt acinar and, in rare cases, ductal and endocrine fates, deviating from the ADM and PanIN courses. While deleting p53 in Lfng-expressing centroacinar cells drastically increased their descendants (Fig. 3N), deletion of both p53 and Lfng reverted this phenotype (Fig. 5P, Q), suggesting that Lfng may be required for the hyperproliferation of p53-deficient centroacinar cells. Indeed, while Ki67 immunostaining were completely negative in *Lfng-RFP/CreER* and *Lfng*^*fl/fl*^*;Lfng-RFP/CreER* pancreas, *p53*^*fl/fl*^*;Lfng-RFP/CreER* pancreas showed Ki67^+^ cells residing in the centroacinar compartment, and *Lfng*^*fl/fl*^*;p53*^*fl/fl*^*;Lfng-RFP/CreER* pancreas had significantly decreased number of Ki67^+^ cells compared to *p53*^*fl/fl*^*;Lfng-RFP/CreER* (Fig. 5R, S). Finally, Lfng deletion had no effect on the number of RFP^+^ cells (Fig. 5P, Q), ruling out the possibility of decreased *Lfng-RFP/CreER* promoter activity in LPKC-Lfng mice.

We also compared precursor lesions in *Kras;Lfng-RFP/CreER* and *Lfng*^*fl/fl*^*;Kras;Lfng-RFP/CreER* mice after tamoxifen induction followed by caerulein treatment. Lfng deletion had no effect on ADM/PanIN formation under this condition (Supplemental Fig. 3A–C). *R26*^*YFP*^*;Kras;Lfng-RFP/CreER* and *R26*^*YFP*^*;Lfng*^*fl/fl*^*;Kras;Lfng-RFP/CreER* mice showed similar YFP and RFP staining, indicating that Lfng deletion did not alter the fate of Lfng-expressing centroacinar cells targeted by Kras^G12D^ alone (Supplemental Fig. 3D–I).

### Notch3 is a functional Notch receptor for the initiation and progression of PDAC originating from Lfng-expressing centroacinar cell

**Fig. 6:**
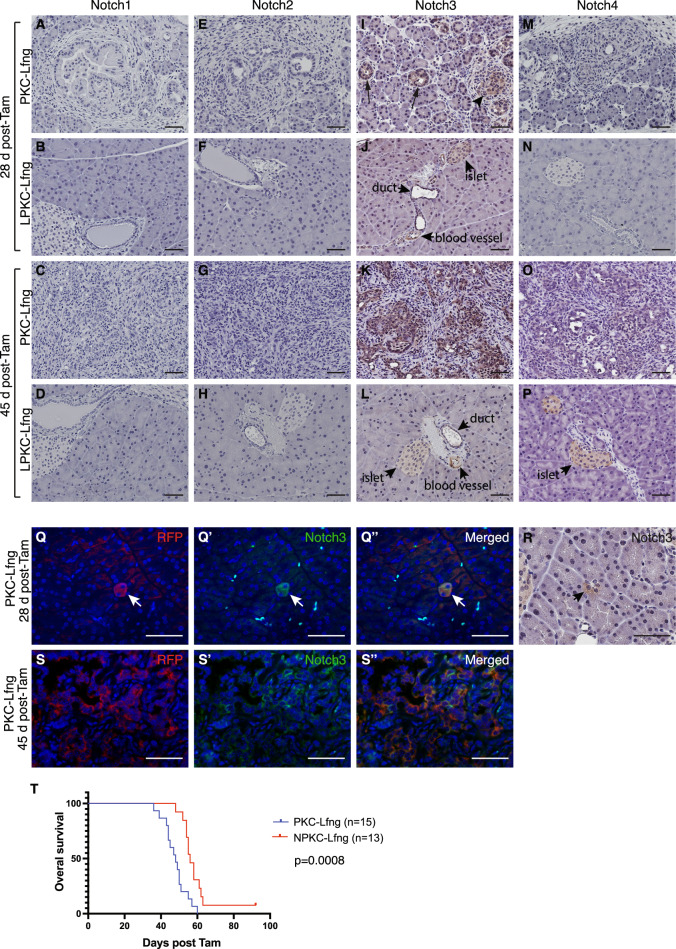
Notch3 is functional in the initiation and progression of PDAC arising from Lfng-expressing cells. **A–D** Immunostaining for Notch1 in the pancreas from *p53*^*fl/fl*^*;Kras;Lfng-RFP/CreER* (PKC-Lfng) and *Lfng*^*fl/fl*^*;p53*^*fl/fl*^*;Kras;Lfng-RFP/CreER* (LPKC-Lfng) mice at 28 and 45 days post tamoxifen injection. **E–H** Immunostaining for Notch2 in the PKC-Lfng and LPKC-Lfng pancreas at 28 and 45 days post-tamoxifen. **I–L** Immunostaining for Notch3 in the PKC-Lfng and LPKC-Lfng pancreas at 28 and 45 days post-tamoxifen. Arrows in I: positive staining in ADM. Arrowhead in I: positive staining in PanIN. **M–P** Immunostaining for Notch4 in the PKC-Lfng and LPKC-Lfng pancreas at 28 and 45 days post-tamoxifen. **Q-Q”** Double immunofluorescence staining for RFP (red) and Notch3 (green) in the PKC-Lfng pancreas at 28 days post-tamoxifen. Arrows: co-staining of RFP and Notch3 in centroacinar cells. **R** Anti-Notch3 immunostaining in the PKC-Lfng pancreas at 28 days post-tamoxifen. Arrow: positive staining in centroacinar cells. **S-S”** Double immunofluorescence staining for RFP (red) and Notch3 (green) in the PKC-Lfng pancreas at 45 days post-tamoxifen. **T** Kaplan-Meier survival analysis for PKC-Lfng and *Nocth3*^*-/-*^*;p53*^*fl/fl*^*;Kras;Lfng-RFP/CreER* (NPKC-Lfng) mice following tamoxifen induction. Log-rank test *p* = 0.0008. Scale bars: 50 μm.

To understand Notch regulation by Lfng during PDAC initiation and progression, we performed immunohistochemistry for all four Notch receptors in the PKC-Lfng and LPKC-Lfng pancreas at 2 time points; e.g., 28 and 45 days post-tamoxifen. Notch1 and Notch2 were undetectable in these mice (Fig. 6A–H). To the contrary, some centroacinar cells (Fig. 6R), all PanIN lesions (Fig. 6I) and tumor cells (Fig. 6K) showed robust Notch3 expression in PKC-Lfng mice. For comparison, Notch3 was detected only in blood vessels and weakly in islets in LPKC-Lfng mice (Fig. 6J, L). Notch4 expression was barely detectable in a few PanIN/PDAC cells in PKC-Lfng mice (Fig. 6M, O), whereas in LPKC-Lfng mice it was completely negative on day 28 post-tamoxifen (Fig. 6N) and weak in islets on day 45 post-tamoxifen (Fig. 6P). Co-expression of Notch3 and RFP was observed in a subset of centroacinar cells (Fig. 6Q-Q”) and in many tumor cells (Fig. 6S-S”) in PKC-Lfng mice. Thus, Notch3 is the sole Notch receptor that is upregulated during tumor initiation and progression from Lfng-expressing centroacinar cells, suggesting an essential role for Lfng-dependent Notch3 signaling in this type of tumor. Indeed, breeding of a *Notch3* null mutation [34] into PKC-Lfng mice significantly prolonged survival (Fig. 6T).

### Lfng exerts an oncogenic role in acinar- and ductal-derived PDACs

**Fig. 7:**
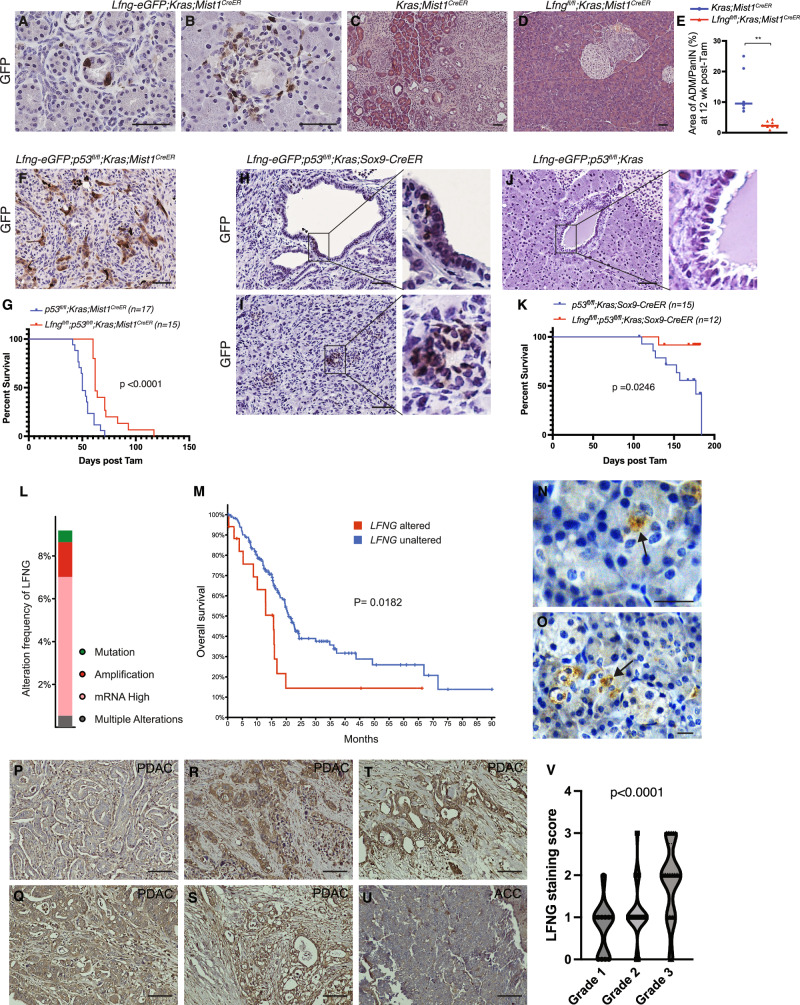
Roles of Lfng in acinar- and ductal-derived pancreatic tumors in mice and association of LFNG expression with tumor grade and survival in human patients. **A**, **B** Anti-GFP immunostaining in the pancreas of *Lfng-eGFP;Kras;Mist1*^*CreER/+*^ mice at 14 weeks post-tamoxifen injection. **C**, **D** Representative photomicrographs of pancreas from *Kras;Mist1*^*CreER/+*^ and *Lfng*^*fl/fl*^*;Kras;Mist1*^*CreER/+*^ mice at 12 weeks after tamoxifen injection. **E** Quantitation of ADM and PanIN lesions depicted in (**C**, **D**), presented as the percentage of total area affected by the precancerous lesions. **p < 0.01. **F** Anti-GFP immunostaining in the pancreas of *Lfng-eGFP****;****p53*^*fl/fl*^*;Kras;Mist1*^*CreER/+*^ mice at 7 weeks post-tamoxifen induction. **G** Kaplan-Meier survival analysis for *p53*^*fl/fl*^*;Kras;Mist1*^*CreER/+*^ and *Lfng*^*fl/fl*^*;p53*^*fl/fl*^*;Kras;Mist1*^*CreER/+*^ mice following tamoxifen injection at 1 month of age. Log-rank test *p* < 0.0001. **H**, **I** Anti-GFP immunostaining in the pancreas of *Lfng-eGFP;p53*^*fl/fl*^*;Kras;Sox9-CreER* mice 6 months after tamoxifen injection. **J** Anti-GFP immunostaining in the pancreas of *Lfng-eGFP;p53*^*fl/fl*^*;Kras* mice. **K** Kaplan-Meier survival analysis for *p53*^*fl/fl*^*;Kras;Sox9-CreER* and *Lfng*^*fl/fl*^*;p53*^*fl/fl*^*;Kras;Sox9-CreER* mice following tamoxifen injection at 1 month of age. Log-rank test *p* = 0.0246. **L** Genetic alterations of *LFNG* in patients with PDAC. **M** Overall survival of PDAC patients with or without *LFNG* alterations. Log-rank test *p* = 0.0182. Data derived from The Cancer Genome Atlas Program (TCGA) pancreatic cancer dataset. **N**, **O** Immunostaining for LFNG in tumor-adjacent near normal tissues from PDAC patients. Arrows: centroacinar cells. **P**–**U** Representative photomicrographs of anti-LFNG immunostaining on a human pancreatic tissue microarray containing specimens of adenocarcinoma (*n* = 38), acinic cell carcinoma (*n* = 1), and squamous cell carcinoma (*n* = 1), duplicate cores per case. ACC: acinic cell carcinoma. **V** Quantitation of LFNG immunostaining in adenocarcinomas of Grade 1, 2, and 3. Staining scores: 0 = completely negative, 1 = weak, 2 = intermediate, and 3 = strong. ANOVA *p* < 0.0001. Scale bars: 50 μm in (**A**–**D**, **F**, and **H**–**J**); 25 μm in (**N**, **O**); 100 μm in (**P**–**U**).

To determine whether Lfng plays any role in acinar cell-derived PDAC, we first crossed a *Lfng-eGFP* reporter into *Kras;Mist1*^*CreER/+*^, an acinar-derived PADC precursor model [32]. GFP expression was detected in very few pancreatic cells with centroacinar location (Supplemental Fig. 4A, B) and in endothelial cells (Supplemental Fig. 4C, D) in *Lfng-eGFP* mice, but was turned on specifically in ADM and PanIN lesions in *Lfng-eGFP;Kras;Mist1*^*CreER/+*^ mice (Fig. 7A, B). Consistent with the Lfng upregulation in precursor lesions, deletion of Lfng decelerated lesion formation in this model (Fig. 7C–E). Notably, pancreas in 2 out of 10 of *Lfng*^*fl/fl*^*;Kras;Mist1*^*CreER/+*^ mice showed complete loss of acini, leaving islets and desmoplasia-associated ducts embedded within a fat pad (Supplemental Fig. 5). Lfng is also upregulated in acinar-derived tumor cells as shown by robust GFP expression in *Lfng-eGFP;p53*^*fl/fl*^*;Kras;Mist1*^*CreER/+*^ mice (Fig. 7F), and deletion of Lfng in *p53*^*fl/fl*^*;Kras;Mist1*^*CreER/+*^ significantly prolonged survival (Fig. 7G). Both *R26*^*YFP*^*;Mist1*^*CreER/+*^ and *R26*^*YFP*^*;Lfng*^*fl/fl*^*;Mist1*^*CreER/+*^ mice showed about 75% recombination efficiency among acinar cells (Supplemental Fig. 6), indicating that suppression of PanIN/PDAC in *Lfng*^*fl/fl*^*;Kras;Mist1*^*CreER/+*^ and *Lfng*^*fl/fl*^*;p53*^*fl/fl*^*;Kras;Mist1*^*CreER/+*^ mice was not due to decreased *Mist1*^*CreER/+*^ activity. Double immunofluorescence staining for GFP and individual Notch receptors in *Lfng-eGFP;p53*^*fl/fl*^*;Kras;Mist1*^*CreER/+*^ mice showed that Notch3 was highly expressed in tumor cells, some of which co-expressing Lfng or adjacent to Lfng-expressing cells (Supplemental Fig. 7A–C). Notch1 and Notch2 were undetectable, whereas Notch4 was noted in limited number of tumor cells (Supplemental Fig. 7D–F). These results suggest an oncogenic role for Lfng through Notch3 in acinar-derived PDAC.

Next, we determined whether Lfng also plays a role in ductal cell-derived PDAC using *Sox9-CreER*-mediated model [35, 36]. Although *R26*^*YFP*^*;Sox9-CreER* pancreas contained very few YFP^+^ ductal cells (data not shown), *R26*^*YFP*^*;p53*^*fl/fl*^*;Kras;Sox9-CreER* pancreas showed YFP staining in normal and tumorous ducts (Supplemental Fig. 8A–D) and in PDAC cells (Supplemental Fig. 8E, F), confirming the ductal origin of PDAC in this model. *Lfng-eGFP;p53*^*fl/fl*^*;Kras;Sox9-CreER* showed GFP in metaplastic ductal cells and tumor cells (Fig. 7H, I), whereas *Lfng-eGFP;p53*^*fl/fl*^*;Kras* pancreas showed no GFP in normal ducts (Fig. 7J). Moreover, deletion of Lfng in *p53*^*fl/fl*^*;Kras;Sox9-CreER* mice significantly delayed tumor onset and improved survival (Fig. 7K). Like acinar-derived PDAC, these tumors also exhibited robust Notch3 expression, co-localizing or juxtaposing with Lfng (Supplemental Fig. 7G–I). These data suggest that Lfng plays an oncogenic role in ductal-derived PDAC as well.

### High LFNG expression is associated with high grade and poor survival in human PDAC

Alterations of *LFNG*, predominantly in the forms of mRNA upregulation and gene amplification, were detected in 9% PDAC patients and were associated with shortened overall survival (Fig. 7L, M). Consistent with its expression in mouse pancreas, LFNG protein was detected in a subset of cells at the centroacinar vicinity in normal and adjacent normal pancreatic tissues from human patients (Fig. 7N, O). Staining for LFNG in a pancreatic tissue microarray showed varied intensity, with the staining score positively correlating with tumor grade in adenocarcinoma specimens (Fig. 7P–T, V). Of note, acinic cell carcinoma of the pancreas stained negative for LFNG (Fig. 7U). These results suggest that LFNG may play an important role in human PDAC, consistent with our investigation in murine model.

## Discussion

Adult pancreas is thought to harbor a dormant progenitor cell population capable of initiating tumor growth under oncogenic stimulation [37]. Studies in mice showed that both acinar and ductal cells of the adult pancreas can be targeted to form PDAC by oncogenic Kras expression when coupled with p53 deletion or point mutation [31, 35, 36]. On the other hand, deletion of Pten has been shown to cause ductal metaplasia putatively derived from centroacinar cells [9]. Here we report for the first time that Lfng-expressing centroacinar cells are readily targeted to form PDAC by Kras^G12D^ when coupled with p53 deletion. Lfng expression in mouse pancreas is overlapping with the centroacinar progenitor marker Aldh1b1. LFNG expression in human PDAC is positively correlated to expression of HES1 [26], which is confined to the terminal duct and centroacinar cells in adult pancreas [38]. Thus, Lfng marks a centroacinar subpopulation serving as a cell-of-origin for PDAC.

In previous studies targeting almost all acinar (or ductal) cells with oncogenic Kras and p53 mutations, only a small subset of targeted cells gave rise to PDAC. How the transforming activity of Kras is constrained and/or which subset of exocrine cells is more susceptible to oncogenic transformation, remains unresolved. Through extensive comparison of tumor development from targeting Lfng-expressing centroacinar cells and Mist1-expressing acinar cells with the same set of Kras and p53 mutations, we showed that Lfng-expressing centroacinar cells are resistant to targeting by Kras^G12D^ alone or in combination with p53^R172H^. In marked contrast, Lfng-expressing centroacinar cells were readily susceptible to oncogenic Kras when coupled with p53 deletion. Deleting p53 in Lfng-expressing centroacinar cells caused drastic expansion of these cells’ descendants. Thus, Lfng marks a subset of centroacinar cells that are tightly controlled by p53, and disruption of normal p53 function may be required for oncogenic transformation of these cells. p53^R172H^ acquires gain of functions that are tumor-promoting and, in combination with Kras^G12D^, is sufficient to drive PDAC in differentiated acinar cells. Although p53^R172H^ mutant protein interferes with the normal function of wild-type p53 protein, the decrease in normal p53 function is not sufficient to drive PDAC in centroacinar cells. Our study therefore identified a centroacinar subpopulation as a unique cell-of-origin for PDAC with p53 deletion. Cell-of-origin is an important determinant of PDAC precursor initiation, disease progression, and molecular subtype [31, 35, 36]. Compared with acinar- and ductal-derived tumors, PDAC derived from Lfng^+^ centroacinar cells was more aggressive, affecting the entire pancreas and leading to worse survival. Noteworthily, centroacinar-targeted pancreas contained high number of Aldh1^+^ cells and apparent acinar (or ductal) cells expressing insulin, suggesting the existence of multipotent cancer stem cell.

Notch controls cell fate specification and differentiation during pancreas organogenesis and in the adult exocrine pancreas [39–42]. Aberrant Notch activation maintains an undifferentiated state, therefore promoting tumor initiation through expansion of an undifferentiated population. PKC-Lfng mice (targeting Lfng-expressing cells with Kras^G12D^ and p53 deletion) showed increased Hes1 expression, indicative of increased Notch activation. Concurrent Lfng deletion reverted the increase in Hes1 expression, suggesting that Lfng might be required for Notch activation in PKC-Lfng mice. By examining the expression of individual Notch receptors in both PKC-Lfng and LPKC-Lfng pancreas tissues, we identified Notch3 as a functional Notch receptor for tumor initiation and progression from Lfng-expressing cells. Notch3 deletion partially suppressed tumor development, likely due to redundancy or compensational upregulation of other Notch receptors. Interestingly, NOTCH3 expression was found upregulated near 6-fold in human PDAC specimens compared to normal pancreas, whereas NOTCH2 and NOTCH4 were only moderately upregulated [5]. Examination in tissue microarray found overexpression of NOTCH3 in 84% and NOTCH4 in 31% of PDACs, whereas NOTCH1 was only detected in blood vessels [43]. High expression of NOTCH3 protein was correlated with tumor grade, invasion, metastasis, and shortened survival [44], whereas low expression of NOTCH3 mRNA was associated with longer survival in patients with unresectable PDAC [45]. The presence of NOTCH3 in tumor nuclei, presumably an indication of activation, was linked to a more aggressive disease phenotype and was associated with reduced survival following tumor resection [46, 47]. These studies suggest a key role for NOTCH3 in a large portion of human PDAC.

We previously reported acceleration of PDAC by *Lfng* deletion in *Kras*^*G12D*^*;Pdx1-Cre* model [10]. Here we show that Lfng deletion suppressed tumor development from all three exocrine lineages in the adult pancreas, supporting an oncogenic role for Lfng under these more physiological conditions. The discrepancy between the tumor-suppressive role of Lfng previously observed and the oncogenic role suggested in this study may be related to the differences in the mouse models, including the developmental stages and the cell types being targeted, as well as the oncogenic mutations employed. In the previous model, Pdx1-Cre is activated in the pancreatic lineage during embryonic stage, resulting in Kras^G12D^ expression in the developing pancreas. Tumors may arise from pancreatic progenitor cells in these mice. Whereas in the current study, centroacinar, acinar, or ductal cells in the adult pancreas were targeted to form PADC. Lfng may act as a tumor suppressor or oncogene depending on the cell-of-origin of PDAC. In addition, since Lfng is co-expressed with Ptf1a during embryonic development, Pdx1-Cre-mediated Lfng deletion may cause defective differentiation and accumulation of pancreatic progenitor cells, which may accelerate tumor development due to an increase in tumor-initiating cells in the *Kras*^*G12D*^*;Pdx1-Cre* model. Finally, in the current study p53 is deleted in all three models targeting exocrine cell types of the adult pancreas. It remains to be determined whether Lfng also plays an oncogenic role in these cell types when p53^R172H^ is involved.

Similar to the deletion of Lfng, deletion of Hes1 also had opposite outcomes in two different PDAC models, being able to promote PDAC in *Kras*^*G12D*^*;Ptf1a* + */Cre* (expressing Kras^G12D^ starting from embryonic stage) [14] while suppressing it in *Trp53*^*R172H*^*;Kras*^*G12D*^*;Ela1-CreERT2* (expressing Kras^G12D^ and Trp53^R172H^ in adult acinar cells) [15]. PDAC may arise from undifferentiated (or poorly differentiated) acinar progenitors in *Kras*^*G12D*^*;Ptf1a* + */Cre* mice. *Ptf1a* + */Cre*-mediated Hes1 deletion caused defective exocrine differentiation with accumulation of acinar progenitors [14], which may contribute to the accelerated tumor development. Conversely, Notch activation and Hes1 upregulation may be required for the reprogramming and oncogenic transformation of acinar cells in *Trp53*^*R172H*^*;Kras*^*G12D*^*;Ela1-CreERT2* mice. These studies underscore differential roles of Notch signaling in PDAC originating from distinct cells-of-origin, and the importance of choosing PDAC models that target mature pancreatic cells rather than the entire pancreatic anlage during embryonic stage.

*LFNG* gene amplification and mRNA upregulation were detected in 9% of PDAC patients, associating with significantly shortened overall survival. High LFNG immunostaining was correlated with advanced tumor stage and pathological grade, poor differentiation, distant metastasis, and poor survival [25], whereas its low expression predicted better overall survival [26]. Secretion of LFNG protein was higher in aggressive PDAC cell line [48], and inactivation of LFNG in Panc-1 impaired proliferation and migration [10, 26]. Collectively, genomic analysis and studies of human pancreatic tissues corroborate our finding in adult mice that Lfng exerts an oncogenic role in PDAC.

## Materials and methods

### Mouse strains and treatments

*Lfng-RFP/CreERT2* obtained from The Jackson Laboratory was originally generated by Dr. McMahon and colleagues (The GenitoUrinary Development Molecular Anatomy Project, 2010). *Lfng-eGFP* resulted from The Gene Expression Nervous System Atlas (GENSAT) Project, The Rockefeller University, has been described previously [22, 23]. Generation of *Lfng*^*flox/flox*^ and *Notch3*^*β-Geo/β-Geo*^ was described elsewhere [34]. All other mouse strains including *Kras*^*LSL-G12D*^, *Trp53*^*flox/flox*^, *Rosa*^*LSL-YFP*^, *Mist1*^*CreERT2*^, and *Sox9-Cre/ERT2* were obtained from The Jackson Laboratory. Animals were treated with tamoxifen (62.5 mg/Kg body weight) on three consecutive days (one dose per day) at 4–6 weeks of age. Caerulein (125 μg/Kg body weight) was administrated daily for 4 weeks via i.p. injection. Approximately equal number of male and female mice were randomly assigned to different groups in all experiments.

### Human specimens

Patient specimens were obtained from the Department of Pathology, the Second Xiangya Hospital during surgery and in routine diagnostic biopsies. All research biopsies were evaluated by pathologists specialized in pancreatic cancer diagnostics and ensured adequate quantity of tumor tissues were used for analysis. Adjacent tissue samples were located within 1 cm of the tumor margin and were confirmed to be noncancerous by pathological examination. A pancreatic cancer tissue array (PA1001D) with adjacent normal and normal pancreas tissue was purchased from TissueArray.Com LLC (Derwood, MD, USA).

### Histology and immunohistochemistry

Mouse tissues were fixed in formalin and processed for paraffin-embedded blocks as per standard procedure. For histology, 5-μm tissue sections were stained with hematoxylin and eosin. For immunohistochemistry, tissue sections were rehydrated, followed by microwave antigen retrieval, and stained according to standard protocol. Photomicrographs of stained tissues were acquired using a Nikon Eclipse 80i microscope with NIS-Elements imaging software. The investigators were blinded to the genotype of the tissue when the photomicrographs were taken. Primary antibodies used for immunostaining were: RFP (Evrogen, Moscow, Russia; AB234), Aldh1a1 (Proteintech, Rosemont, IL, USA; 60171-1-lg), Aldh1b1 (Proteintech, 15560-1-AP), YFP/GFP (ThermoFisher, Waltham, MA, USA; A-11122), Insulin (Cell Signaling, Danvers, MA, USA; C27C9, #3014), CK19 (DSHB, University of Iowa; TROMA-III), E-Cadherin (Cell Signaling, #3195), Vimentin (Cell Signaling, #5741), Cleaved caspase 3 (Cell Signaling, #9661), Amylase (MilliporeSigma, Burlington, MA, USA; A8273), Notch1 (Cell Signaling, #3608), Notch2 (DSHB, University of Iowa, C651.6DbHN), Notch3 (ProteinTech, 55114-1-AP), Notch4 (MilliporeSigma, 09-089), LFNG (Abcam, Waltham, MA, USA; ab192788), and a Rabbit anti-LFNG mAb produced by AbMax Biotechnology (Beijing, China) using the peptide CPPPLPAERGRRALR as antigen.

### X-gal staining

Mouse pancreas was fixed in fixative solution (1.0% formaldehyde, 0.02% Nonidet P-40 in PBS), washed with washing buffer (2 mM MgCl2, 0.02% Nonidet P-40 in PBS), saturated with 30% sucrose, then embedded in Tissue-Tek O.C.T. compound (Sakura Finetek, Torrance, CA, USA) for frozen sections. Pancreas sections were stained with X-gal for 6 h and counterstained with 0.5% eosin.

### Flow cytometry

Pancreas tissue was minced and digested in 2 ml of collagenase type IV (1 mg/ml, StemCell Technology, Vancouver, Canada) plus 2 ml of BSA (10%) at 37 ^o^C for 30 min. Dissociated cell-tissue mixture were pelleted at 300 × *g* for 4 min, and resuspended in 1 ml of HF buffer (HBSS with 1% FBS) plus 4 ml of 0.8% NH4Cl (StemCell Technology) at 4 ^o^C for 10 min. The dissociated cells were pelleted again and resuspended in prewarmed dispase (2 ml, 1 U/ml, StemCell Technology) and DNase I (200 μl, 1 mg/ml, StemCell Technology) for 2 min with gentle mixing. The digestion was stopped by adding 10 ml cold HF buffer. The mixture was passed through a 40 μm mesh strainer to obtain the single cell suspension. After adjusting the cell concentration to 10^7/ml, pancreatic epithelial cells were enriched using EasySep mouse epithelial cell enrichment kit II (StemCell Technology) according to the manufacturer’s instruction. Flow cytometry was performed by standard procedures with the following antibodies: PE-Cyanine7 rat anti-CD44 (Thermofisher) and Alexa Fluor 488 mouse anti-CD24 (Biolegend, San Diego, CA, USA). Fluorescence was recorded using Gallios Flow Cytometer (Beckman Coulter, Indianapolis, IN, USA) and analyzed with Kaluza flow cytometry analysis software.

### Quantitative reverse transcription PCR

Total RNA was prepared from mouse pancreas tissue using RNeasy Mini kit (Qiagen, Germantown, MD, USA) and reverse-transcribed using iScript cDNA synthesis kit (Bio-rad, Hercules, CA, USA). Real-time PCR was performed using QuantiTect SYBR Green PCR Kit (Qiagen) with BioRad CFX96 qPCR system. Primer sequences for *Hes1* was as previously reported [49]. Results from triplicate PCR were normalized with the expression level of Gapdh using the equation 2^-Δ*C*T^, where Δ*C*T = *C*T_Hes1_ – *C*T_Gapdh_.

### Analysis of human pancreatic cancer data set

The Pancreatic Adenocarcinoma data set (TCGA, Firehose Legacy; *n* = 186) hosted in cBioPortal (https://www.cbioportal.org) was used for analysis of genetic alterations in *LFNG*. Patient survival related to *LFNG* alterations was analyzed using the same data set and online tools in cBioPortal.

### Statistical analysis

All statistical analyses were performed using GraphPad Prism version 10.1.1 (GraphPad, San Diego, CA). Each experiment was independently repeated three times. The exact sample size *(n)* in each group for survival analysis is provided in figures. Data were presented as the mean with individual data points. Statistical significance was assessed using two-tailed Student’s *t* test for two-group comparison or one-way ANOVA for multiple group comparison. Animal survival was calculated by the Kaplan-Meier method and compared by nonparametric log-rank test. *P* value of 0.05 or less was considered statistically significant.

## Supplementary information

Supplemental Figure Legends

Supplemental Figure 1

Supplemental Figure 2

Supplemental Figure 3

Supplemental Figure 4

Supplemental Figure 5

Supplemental Figure 6

Supplemental Figure 7

Supplemental Figure 8

## Data Availability

All data associated with this study are presented in the paper or Supplementary Information. Requests for further information should be directed to corresponding author.
