# Supplementary material for: Lfng-expressing centroacinar cell is a unique cell-of-origin for p53 deficient pancreatic cancer
Source: Oncogene. 2024 Nov 15;44(6):348–62. doi: 10.1038/s41388-024-03226-7 (PMC11790384; doi:10.1038/s41388-024-03226-7)
Supplement: Supplementary file 1 — Supplemental Figure Legends [file 41388_2024_3226_MOESM1_ESM.docx]

**SUPPLEMENTAL FIGURE LEGENDS**

**Supplemental Fig. 1 Effect of caerulein treatment on tumor development and survival of PKC mice. A** Kaplan-Meier survival analysis for *p53^fl/fl^;Kras;Lfng-RFP/CreER* (PKC-Lfng) mice following 3 doses of tamoxifen between day 30 and 32 after birth, with or without one month of daily caerulein treatment starting from day 35. Log-rank test p=0.8947. **B** Representative photomicrographs of pancreas from PKC-Lfng mice at 3 months post-tamoxifen induction (with caerulein treatment) as described in A. Scale bars: 100 μm. **C** Kaplan-Meier survival analysis for PKC-Lfng mice following 3 doses of tamoxifen between day 30 and 32 after birth, with or without one month of daily caerulein treatment starting from day 60. Log-rank test p=0.7986.

**Supplemental Fig. 2 Oncogenic transformation of Lfng-expressing cells by combination of *p53^R172H^* and *Kras^G12D^* mutations.** **A-C** Representative photomicrographs of pancreas from *p53^R172H^;Kras;Lfng-RFP/CreER* mice at 20 weeks after tamoxifen administration at 4 weeks of age. **D-F** Representative histology of pancreatic tumors from *p53^R172H^;Kras;Lfng-RFP/CreER* mice receiving tamoxifen injection at 4 weeks of age followed by 4 weeks of daily treatment with caerulein. **G** Quantitation of pancreatic lesions in *p53^R172H^;Kras;Lfng-RFP/CreER* mice depicted in A-F, presented as the percentage of total area affected by ADM, PanIN, and PDAC. ** p<0.01. **H** Kaplan-Meier survival analysis for *p53^R172H^;Kras;Lfng-RFP/CreER* mice following tamoxifen administration, with or without caerulein treatment. Log-rank test p=0.0146. **I-K** Anti-YFP immunostaining in the pancreas from *R26^YFP^;p53^R172H^;Kras;Lfng-RFP/CreER* mice following tamoxifen injection and caerulein treatment. **L-N** Anti-RFP immunostaining in the pancreas from *R26^YFP^;p53^R172H^;Kras;Lfng-RFP/CreER* mice following tamoxifen injection and caerulein treatment. Scale bars: 100 μm in A-F; 25 μm in I-N.

**Supplemental Fig. 3 Deletion of Lfng does not suppress precancerous lesions induced by Kras^G12D^ coupled with caerulein treatment. A,B** Representative photomicrographs of pancreas from *Kras;Lfng-RFP/CreER* and *Lfng^fl/fl^;Kras;Lfng-RFP/CreER* mice at 20 weeks of age. These mice were treated with three doses of tamoxifen at 4 weeks of age, followed by 4 weeks of daily treatment with caerulein. **C** Quantitation of precancerous lesions in *Kras;Lfng-RFP/CreER* and *Lfng^fl/fl^;Kras;Lfng-RFP/CreER* mice depicted in A and B, presented as the percentage of total area affected by PanIN and PDAC lesions. ns: non-significance. **D,E** Anti-YFP immunostaining in the pancreas from *R26^YFP^;Kras;Lfng-RFP/CreER* and *R26^YFP^;Lfng^fl/fl^;Kras;Lfng-RFP/CreER* mice at 16 weeks of age. These mice were treated with three doses of tamoxifen at 4 weeks of age, followed by 4 weeks of daily treatment with caerulein. **F-I** Anti-RFP immunostaining in the pancreas from *R26^YFP^;Kras;Lfng-RFP/CreER* and *R26^YFP^;Lfng^fl/fl^;Kras;Lfng-RFP/CreER* mice at 16 weeks of age, with the same treatment described in D and E. Arrows: positive staining in centroacinar cells (F,G) and in ADM (H) and PanIN (I) lesions. Scale bars: 100 μm in A and B; 50 μm in D-I.

**Supplemental Fig. 4 Lfng expression in the pancreas shown by Lfng-eGFP reporter. A-D** Representative photomicrographs of anti-GFP immunostaining in the pancreas from *Lfng-eGFP* mice at 18 weeks of age. Arrows: positive staining in centroacinar cells (A and B) and endothelial cells (C and D)*.* Scale bars: 25 μm.

**Supplemental Fig. 5 Acinar cell loss and desmoplasia in *Lfng^fl/fl^;Kras;Mist1^CreER/+^* mice. A-D** Representative photomicrographs of pancreas from *Lfng^fl/fl^;Kras;Mist1^CreER/+^* mice at 4 months of age following tamoxifen administration at 1 month of age. Scale bars: 100 μm.

**Supplemental Fig. 6 Deletion of Lfng has no effect on *Mist1^CreER/+^-*mediated recombination in the pancreas. A** Anti-YFP immunostaining in the pancreas from *R26^YFP^;Mist1^CreER/+^* and *R26^YFP^;Lfng^fl/fl^;Mist1^CreER/+^* mice 6 weeks after tamoxifen administration. Scale bars: 50 μm. **B** Quantitation of YFP^+^ cells in the pancreas of *R26^YFP^;Mist1^CreER/+^* and *R26^YFP^;Lfng^fl/fl^;Mist1^CreER/+^* mice depicted in A, presented as the percentage of YFP^+^ cells among all cells in the acinar compartment. ns: non-significance.

**Supplemental Fig. 7 Notch3 expression in acinar- and ductal-derived pancreatic tumors. A-C** Double immunofluorescence staining for GFP and Notch3 in *Lfng-eGFP;p53^fl/fl^;Kras;Mist1^CreER/+^* pancreatic tumors. Arrowheads: a tumor cell co-expressing GFP and Notch3 (A) and GFP^+^ cells adjacent to Notch3-expressing cells (B and C). **D-F** Double immunofluorescence staining for GFP and Notch1 (D), GFP and Notch2 (E), GFP and Notch4 (F) in *Lfng-eGFP;p53^fl/fl^;Kras;Mist1^CreER/+^* pancreatic tumors. **G-I** Double immunofluorescence staining for GFP and Notch3 in *Lfng-eGFP;p53^fl/fl^;Kras;Sox9-CreER* pancreatic tumors. Arrowheads: tumor cells co-expressing GFP and Notch3 (G) and GFP^+^ cells adjacent to Notch3-expressing cells (H and I). Scale bars: 25 μm.

**Supplemental Fig. 8 Tracing of ductal cells targeted by Kras^G12D^ expression and p53 deletion under Sox9-CreER. A-F** Representative photomicrographs of anti-YFP immunostaining in pancreas from *R26^YFP^;p53^fl/fl^;Kras;Sox9-CreER* mice 5 months after tamoxifen administration. Arrowheads point to YFP^+^ cells in the normal ducts. Scale bars: 50 μm.
