# Supplementary figures and images for: Lfng-expressing centroacinar cell is a unique cell-of-origin for p53 deficient pancreatic cancer
Source: Oncogene. 2024 Nov 15;44(6):348–62. doi: 10.1038/s41388-024-03226-7 (PMC11790384; doi:10.1038/s41388-024-03226-7)

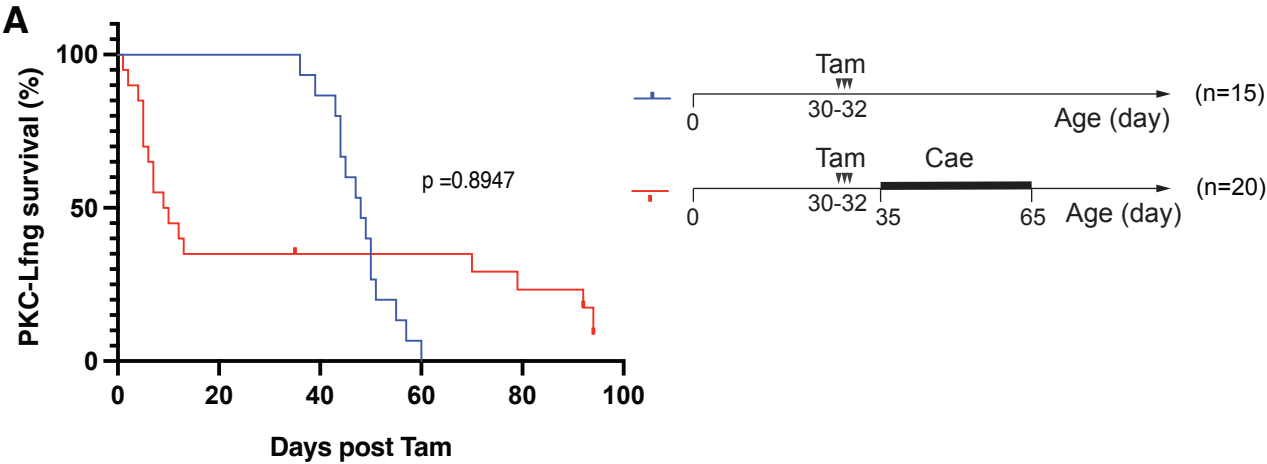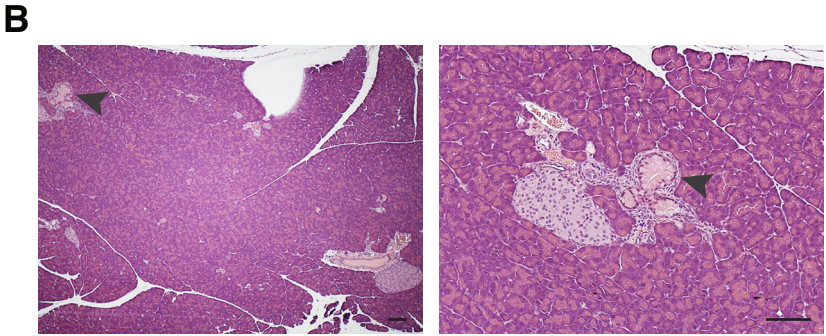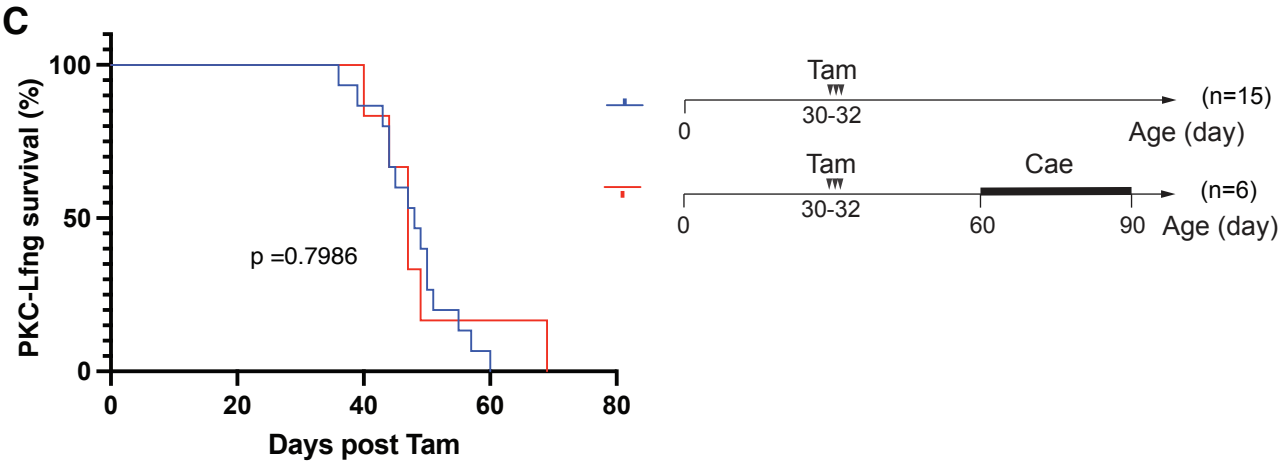

Supplement: Supplementary file 2 — Supplemental Figure 1 [file 41388_2024_3226_MOESM2_ESM.pdf]

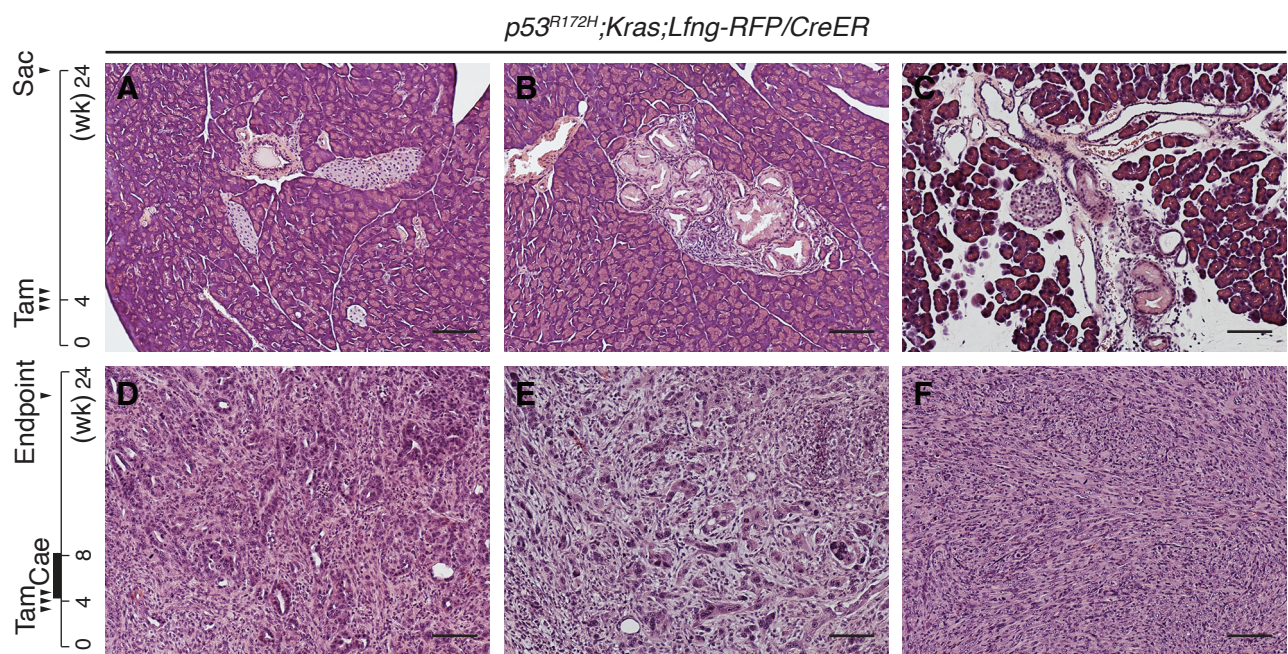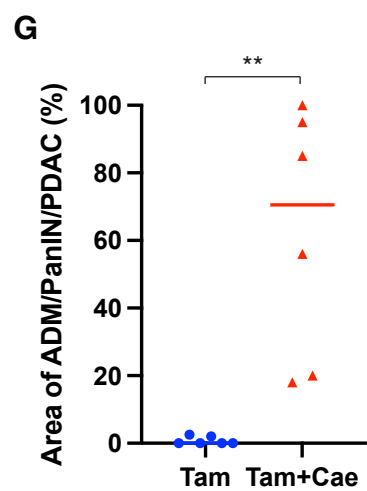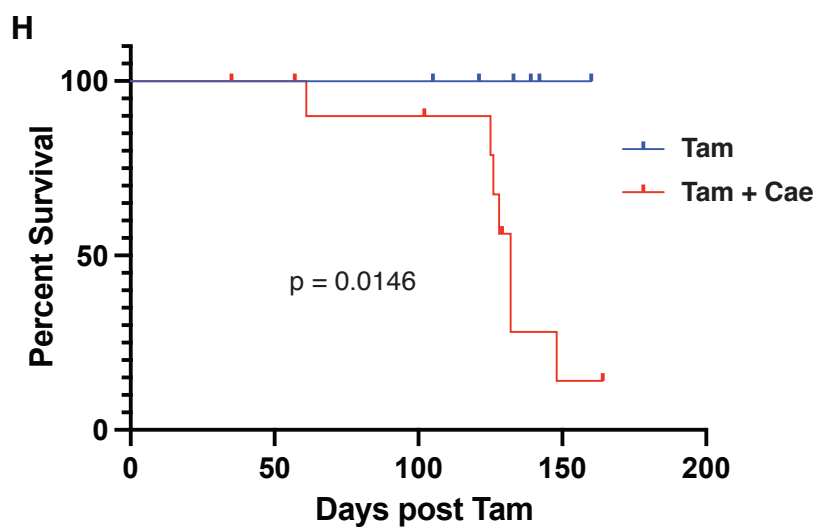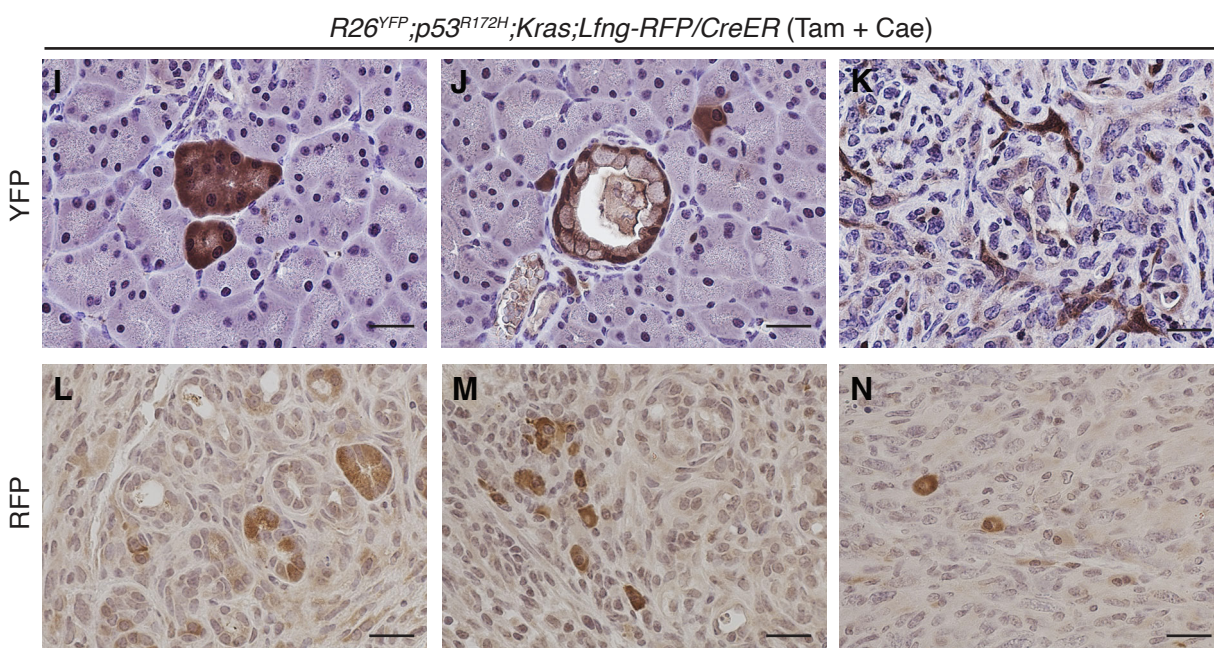

Supplement: Supplementary file 3 — Supplemental Figure 2 [file 41388_2024_3226_MOESM3_ESM.pdf]

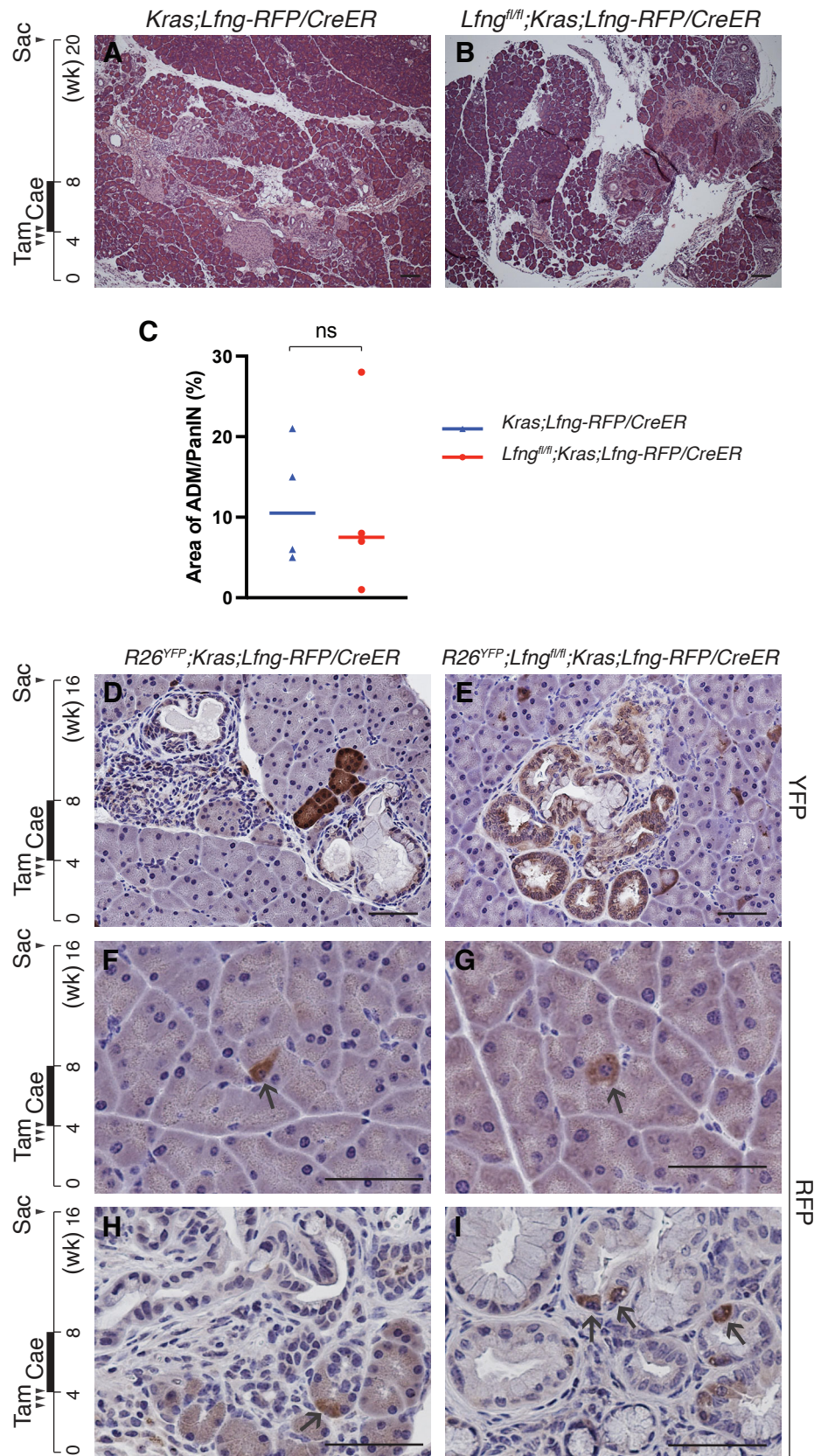

Supplement: Supplementary file 4 — Supplemental Figure 3 [file 41388_2024_3226_MOESM4_ESM.pdf]

*Lfng-eGFP*

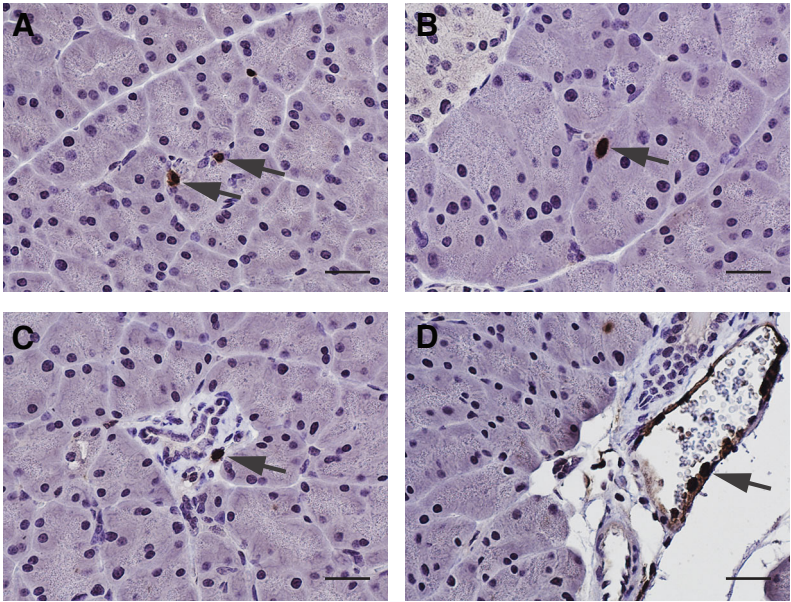

Supplement: Supplementary file 5 — Supplemental Figure 4 [file 41388_2024_3226_MOESM5_ESM.pdf]

Chung\_Supplemental Fig5

*Lfng<sup>fl/fl</sup>;Kras;Mist1<sup>CreER</sup>*

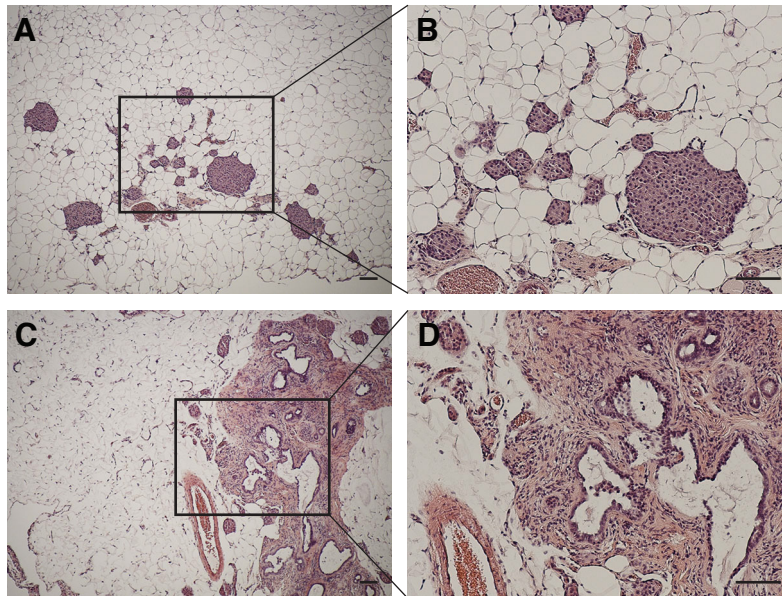

Supplement: Supplementary file 6 — Supplemental Figure 5 [file 41388_2024_3226_MOESM6_ESM.pdf]

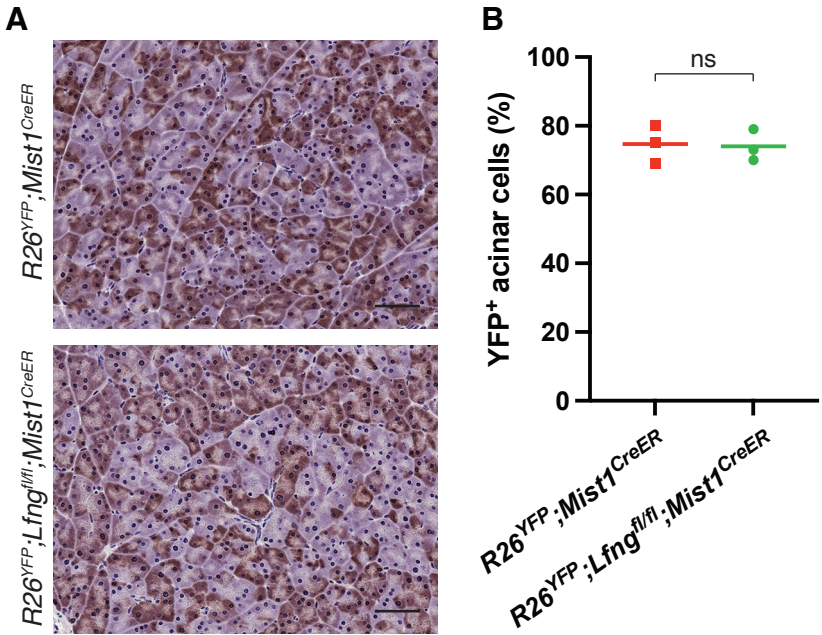

Supplement: Supplementary file 7 — Supplemental Figure 6 [file 41388_2024_3226_MOESM7_ESM.pdf]

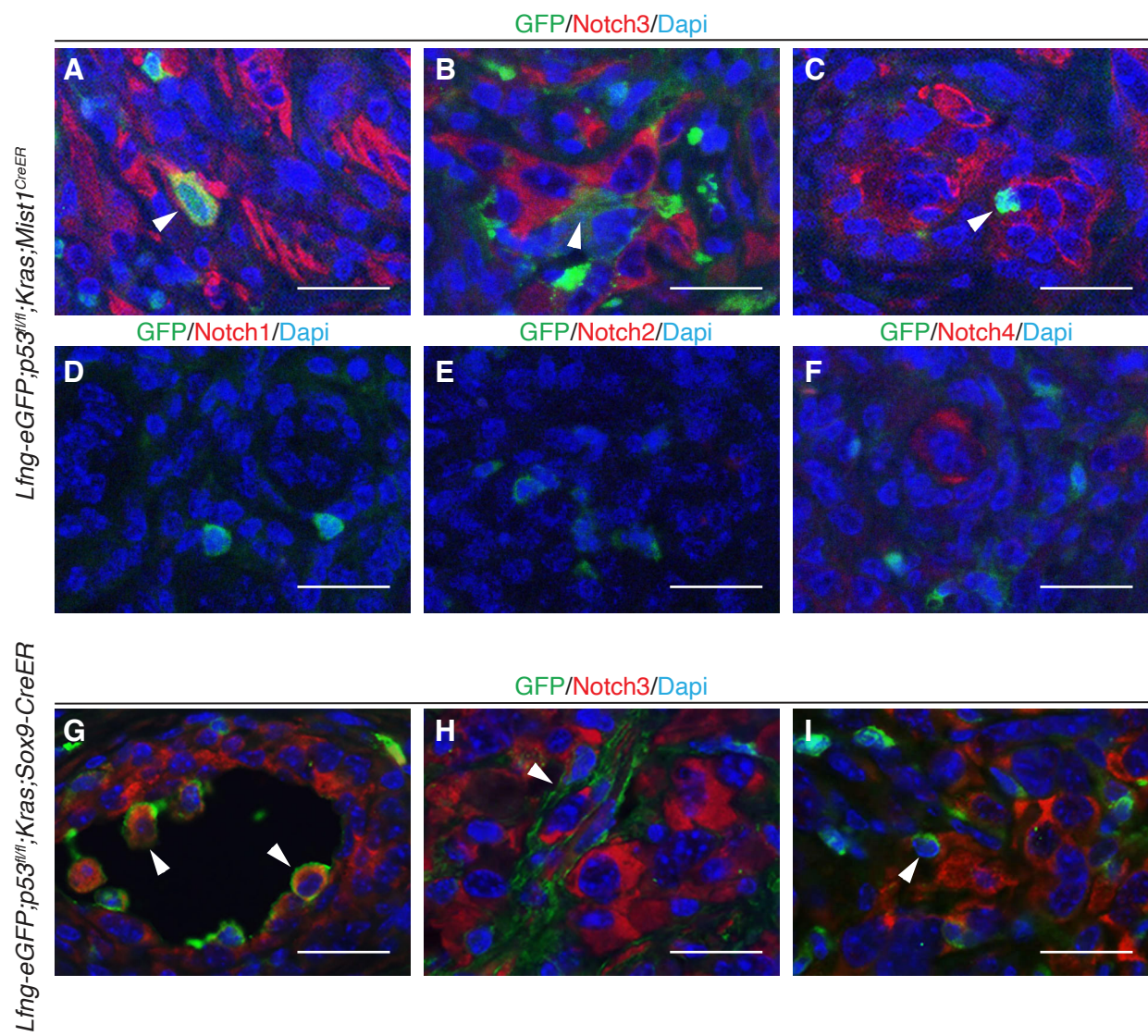

Supplement: Supplementary file 8 — Supplemental Figure 7 [file 41388_2024_3226_MOESM8_ESM.pdf]

*R26<sup>YFP</sup>;p53<sup>fl/fl</sup>;Kras;Sox9-CreER*

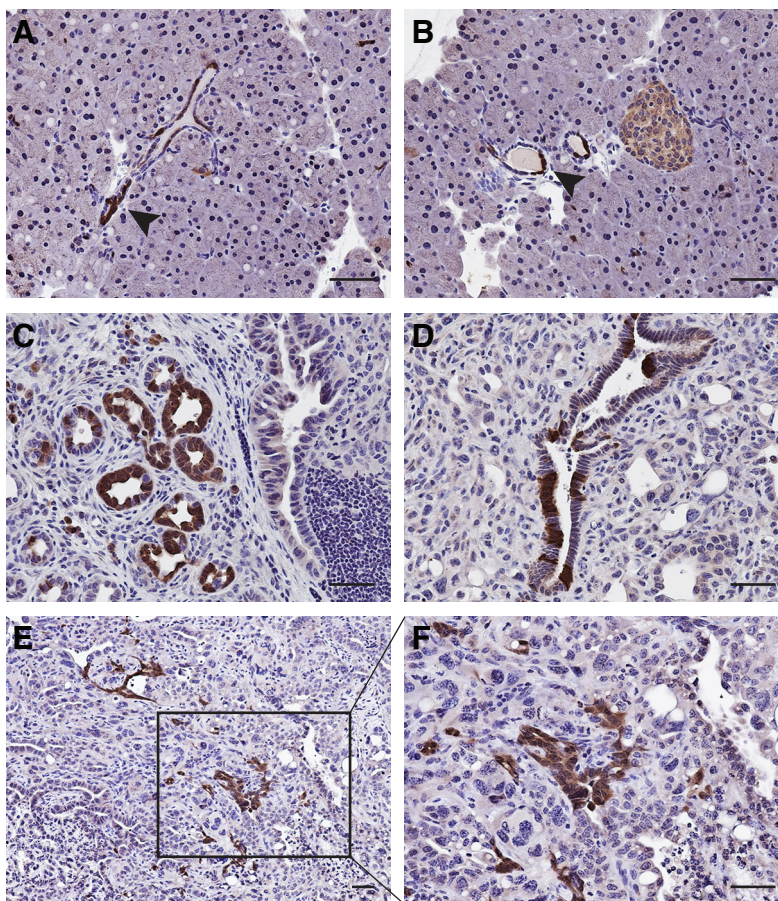

Supplement: Supplementary file 9 — Supplemental Figure 8 [file 41388_2024_3226_MOESM9_ESM.pdf]
